# Chemical Constituents of *Euphorbia stracheyi* Boiss (Euphorbiaceae)

**DOI:** 10.3390/metabo13070852

**Published:** 2023-07-15

**Authors:** Hui Zhu, Xiangxiang Ren, Yanbo Huang, Tao Su, Lei Yang

**Affiliations:** 1 Co-Innovation Center for Sustainable Forestry in Southern China, College of Biology and the Enviroment, Nanjing Forestry University, Nanjing 210037, China; zh8201711509@njfu.edu.cn (H.Z.); renxiangxiang@njfu.edu.cn (X.R.); sutao@njfu.edu.cn (T.S.); 2 Shanghai Key Laboratory of Plant Functional Genomics and Resources, Shanghai Chenshan Botanical Garden, Shanghai 201602, China; huangyanbo@csnbgsh.cn

**Keywords:** *Euphorbiaceae*, *Euphorbia stracheyi*, lathyrane diterpenoid, chemical constituents, X-ray diffraction analysis

## Abstract

*Euphorbia stracheyi* Boiss was used for hemostasis, analgesia, and muscular regeneration in traditional Chinese medicine. To study the chemical constituents of *E. stracheyi*, the ethyl acetate part of the methanol extract of the whole plant was separated by silica gel, sephadex LH-20 column chromatography, and semi-preparative HPLC. The isolation led to the characterization of a new lathyrane type diterpenoid, euphostrachenol A (**1**), as well as eleven known compounds (**2**–**11**), including a lathyrane, three ingenane-type and two abietane-type diterpenoids, two ionones, and two flavonoids. The structures of these compounds were established using 1D- and 2D-NMR experiments, mass spectrometry, and X-ray crystallographic experiments. The MTT method was used to determine the cytotoxic activity of five cancer cell lines (Leukemia HL-60, lung cancer A-549, liver cancer SMMC-7721, breast cancer MCF-7, and colon cancer SW480) on the isolated compounds. However, only compound **4** showed moderate cytotoxicity against these cell lines, with IC_50_ values ranging from 10.28 to 29.70 μM, while the others were inactive. Our chemical investigation also confirmed the absence of jatrophane-type diterpenoids in the species, which may be related to its special habitat.

## 1. Introduction

With nearly 2000 species, the genus *Euphorbia* (*Euphorbiaceae*) is the third largest genus in flowering plants, second only to *Astragalus* (*Fabaceae*) and *Psychotria* (*Rubiaceae*) [1]. Plants of *Euphorbia* are a rich source of structurally diverse macrocyclic and polycyclic diterpenoids, with more than twenty skeleton types, including casbane, jatrophane, daphnane, tigliane, lathyrane, myrsinane, premyrsinane, cyclomyrsinane, paraliane, pepluane, and ingenane [2,3]. The frameworks of these diterpenoids are further modified by different functional group substitutions at different positions and numbers, such as hydroxyl, epoxy, ether, acyl, polyester, and carbonyl groups, which makes the structure of these compounds even more complex and diversified. So far, more than 700 diterpenoids have been isolated from the genus *Euphorbia*, showing anti-tumor, multi-drug-resistance-reversing, anti-inflammatory, antibacterial, and pesticidal activities [2,3]. In 2012, the approval of ingenol 3-angelate (ingenol mebutate) by the U.S. Food and Drug Administration and European Medicines Agency for the treatment of actinic keratosis, a precancerous skin condition, attracted more interest in these types of diterpenoids [4].

Plant secondary metabolites are often lineage-specific, and the occurrence and content of secondary metabolites in a certain plant species could be determined by genetic and environmental factors. Thus, phylogenetically related plants in different habitats may harbor distinctive secondary metabolite profiles. For example, *Salvia officianlis* L. and *S. miltiorrhiza* are native to the Mediterranean and East Asia, respectively. While the former mainly contains tricyclic diterpenoids, the latter accumulates *nor*-diterpene quinones named tanshinones [5]. These differences could influence their pharmacological performances, since both species are medicinally highly valuable. Furthermore, the availability of closely related species with different secondary metabolite constituents allows us to elucidate the biosynthetic pathway of these natural products through a comparative genomics analysis [6]. Clearly, this phenomenon is important and should be given much attention in phytochemical research.

**Figure 1:**
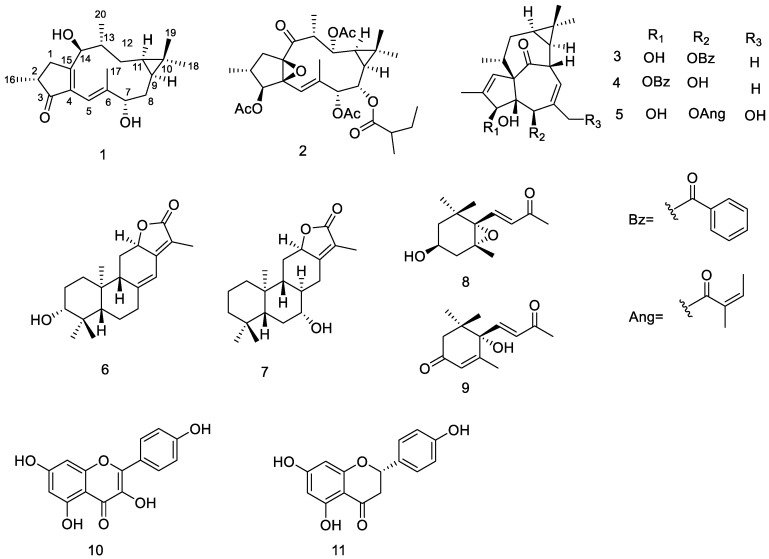
Structures of isolated compounds from *E. stracheyi*.

*Euphorbia stracheyi* Boiss is a perennial noxious weed mainly distributed in the western region of China, including Qinghai, Tibet, Sichuan, and Yunnan province [7]. While the near-cosmopolitan nature of *Euphorbia* is well acknowledged, *E. stracheyi* is one of the few plants that can grow in high-altitude alpine meadow regions (from 1000 to 4900 ASL). *E. stracheyi* roots were used as traditional medicine for hemostasis, analgesia, and muscular regeneration [6]. As such, extensive phytochemical investigations have been performed on this plant, which involved the isolation of flavonoids, coumarins, phenylpropanoids, ionones, steroids, triterpenoids, and its main components, diterpenoids [8,9,10,11,12,13,14]. The cytotoxic activity of the isolated compounds has been evaluated [10,11,13]. The structures of some common compounds are shown in Figure 1. Interestingly, although jatrophane-type diterpenoids are widely occurring in plants of *Euphorbia*, to date there are no compounds of this type isolated from the species. In order to further study the chemical composition of the species, the methanol extract of the whole plant of *E. stracheyi* was investigated in the present study, which led to the isolation and characterization of a new lathyrane diterpenoid, which was designated euphostrachenol A (**1**), together with eleven known compounds, including a lathyrane (**2**), three ingenane (**3**–**5**) and two abietane diterpenoids (**6**, **7**), two ionones (**8**, **9**), and two flavonoids (**10**, **11**). The structures of the isolated compounds were determined through extensive spectroscopic analysis and X-ray crystallographic experiments. In the present study, we report the isolation, structure elucidation, and in vitro cytotoxic activity of these compounds.

## 2. Materials and Methods

### 2.1. Instruments and Materials

X-ray crystallographic analyses was performed on a Bruker APEX DUO X-ray single crystal diffractometer (Bruker, Rheinstetten, Germany). Optical rotations were recorded on a Jasco P1020 spectropolarimeter. IR spectra were measured on a NICOLET iS107 FTIR spectrometer (Thermo Fisher, Massachusetts, USA). NMR spectra were obtained on Bruker AVANCE III 500 MHz and AV 600 MHz superconducting NMR spectrometers (Bruker, Rheinstetten, Germany) with TMS as the internal standard. High-resolution MS data were obtained on an Agilent 1290 UPLC/6540 Q-TOF mass spectrometer in positive mode. UV spectroscopic data were recorded on a Shimadzu-210A double-beam spectrophotometer. Agilent 1290 UPLC/6540 Q-TOF (Agilent, Santa Clara, USA) was used to undertaken liquid chromatography/quadrupole time-of-flight mass spectrometry data.

Semi-preparations were performed on a high-performance liquid chromatograph (HPLC, Agilent 1260 series) using a SunFire-C18 column (5 μm, 10 mm × 250 mm) and an X Select HSS T3 (5 μm, 10 mm × 150 mm) column. Silica gel (100–200, 200–300, and 300–400) (Qingdao Ocean Chemical Co., Ltd., Qingdao, China), Lichroprep RP-18 (40–63 μm, Fuji, Tokyo, Japan), MCI gel CHP-20P (75–150 mm, Mitsubishi Chemical Corp., Tokyo, Japan), and Sephadex LH-20 (20–150 μm, Pharmacia, Shanghai, China) were used for column chromatography. The chromogenic agent was 8% ethanol sulfate, sprayed and heated appropriately for color development.

The whole plants of *E. stracheyi* (flowering) were collected in August 2020 from Gongshan County, Nujiang Prefecture, Yunnan Province. The plant sample was identified by Mr. Yan-Bo Huang of Shanghai Chenshan Botanical Garden. The age of the selected plants was two years, and the material was dried and extracted with methanol.

The sources of the strains used in this study were as follows: Leukemia HL-60, purchased from ATCC (item number: CCL-240, Tissue: Peripheral blood); Lung cancer cells A549, purchased from ATCC (Item number: CRM-CCL-185, Tissue: Lung); Breast cancer MDA-MB-231, purchased from ATCC (item number: CRM-HTB-26, Tissue: Breast); Intestinal cancer cell SW480, purchased from ATCC (item number: CCL-228, Tissue: Colon); Hepatocellular carcinoma cells SMMC-7721, purchased from BNCC, (item number: BNCC338089, Tissue: Liver). ATCC is an American-type culture collection.

### 2.2. Extraction and Separation

The crude extract was obtained by extracting the ion from powdered and air-dried whole plants of *E. stracheyi* (7.0 kg) with methanol three times at room temperature and then combining to concentrate in vacuo. The extract was suspended in water and further extracted sequentially with petroleum ether and ethyl acetate to afford the 700 g ethyl acetate fraction by evaporation. The obtained ethyl acetate extract was then chromatographed on a silica gel column and eluted successively with a gradient of petroleum ether/ethyl acetate (100:0 to 0:100, *v*/*v*) to provide 8 fractions, F1−F8, in which F4, F5, and F6 were further separated due to the richness of compounds in our analysis. Fraction F4 (43 g) was chromatographed on an MCI gel column and eluted with MeOH/H_2_O (40:60 to 100:0, *v*/*v*) to afford fractions F4.1–F4.7. The resulting fraction F4.5 (6.3 g) was chromatographed on a Sephadex LH-20 column (MeOH/MeCl_3_, 1:1, *v*/*v*) to yield three fractions, F4.5.1–F4.5.3. Silica gel column chromatography was used to separate the F4.5.3 fraction (3.6 g) by eluting with a gradient of ether/ethyl acetate (20:1 to 1:1, *v*/*v*) to give 12 subfractions. Subfraction F4.5.3.3 (96.3 mg) was subjected to preparative HPLC separation (CH_3_CN/H_2_O, 70:30, *v*/*v* 3 mL/min) to yield compound **2** (tR = 23.6 min, 4.1 mg). Fraction F4.5.3.10 (605 mg) was purified by preparative HPLC separation (CH_3_CN/H_2_O, 70:30, *v*/*v* 3 mL/min) to yield compound **4** (tR = 26.7 min, 8.8 mg). An MCI gel column was used with a gradient of MeOH/H_2_O (40:60–100:0, *v*/*v*) to separate fraction F5 (53 g), which led to 12 fractions. F5.8 (6.5 g) was further purified by silica gel column chromatography and eluted with an ether/ethyl acetate gradient of (20:1–1:1, *v*/*v*) to afford 6 fractions, F5.8.1–F5.8.6. The obtained fraction F5.8.2 (1.5 g) was separated by a Sephadex LH-20 column (MeOH/MeCl_3_ 1:1, *v*/*v*) to afford 4 fractions. Fraction F5.5.2.4 (29.3 mg) was subjected to preparative HPLC separation with gradients of 82:18 (CH_3_CN/H_2_O, *v*/*v* 3 mL/min) to yield compound **3** (tR = 26.7 min, 5.7 mg). Fraction F6 (61 g) was loaded onto an MCI gel column with a MeOH/H_2_O gradient of (40:60–100:0, *v*/*v*), resulting in 10 fractions, in which fraction F4.7 was found to be pure compound **1** (17.8 mg). Fraction F6.6 (10 g) was subjected to the silica gel column with an ether/ethyl acetate gradient of (20:1–1:1, *v*/*v*) to yield 6 fractions, in which fraction F6.5 (1.1 g) was subjected to a Sephadex LH-20 column (MeOH/MeCl_3_ 1:1, *v*/*v*) to give 9 fractions F6.5.1–F6.6.9. Then, fraction F6.5.2 (163.0 mg) was separated by silica gel column chromatography with an ether/ethyl acetate gradient of (10:1–2:1) to yield compounds **8** (9.9 mg) and **9** (1.9 mg). The fraction F6.5.4 (0.482 g) was chromatographed on a silica gel column with an ether/ethyl acetate gradient of (20:1–1:1, *v*/*v*) to afford compounds **10** (8.5 mg) and **11** (13 mg). The fraction F6.8 (7.3 g) was separated with a Sephadex LH-20 column (MeOH/MeCl_3_ 1:1, *v*/*v*) to give six fractions, in which fraction F6.8.3 (695 mg) was further separated by silica gel column chromatography with a ether/ethyl acetate gradient of (10:1–5:1, *v*/*v*) to afford 8 fractions. The resulting fraction F6.8.3.6 (51.1 mg) was purified by preparative HPLC (CH_3_CN/H_2_O, 60:40, *v*/*v* 3 mL/min) to give compounds **6** (tR = 14.4 min, 11.9 mg) and **7** (tR = 15.3 min, 6.9 mg). The fraction F6.8.3.7 (12.3 mg) was then purified by preparative HPLC (CH_3_CN/H_2_O, 77:23, *v*/*v* 3 mL/min) to give compound **5** (tR = 14.8 min, 11.2 mg).

### 2.3. X-ray Crystallographic Analyses

A light, colorless crystal of **1** was used for the X-ray crystallographic analysis on a Bruker APEX DUO diffractometer equipped with Cu Kα radiation (λ = 1.54178 Å). To solve and determine the structures and absolute configurations of **1**, the ShelXT 28 program with Intrinsic Phasing was used [15].

The crystallographic data of Compound **1** is listed as follows: M = 318.44, approximate dimensions 0.800 × 0.160 × 0.110 mm^3^, calculated density 1.189 Mg/m^3^, a = 10.0464(12) Å, b = 9.5021(11) Å, c = 10.0557(12) Å, α = 90°, β = 112.043(4)°, γ = 90°, V = 889.77(18) Å3, T = 100.(2) K, space group P1211, Z = 2, 17,575 reflections measured, 3416 independent reflections (Rint = 0.0553). The final R1 values were 0.0341 (I > 2σ(I)). The final wR(F2) values were 0.0848 (I > 2σ(I)). The final R1 values were 0.0359 (all data). The final wR(F2) values were 0.0866 (all data). The goodness of fit on F2 was 1.048. Flack parameter = 0.03(9).

Crystallographic data for the structure of compound **1** have been deposited at the Cambridge Crystallographic Data Center (https://ccdc.cam.ac.uk, accessed on 9 May 2023) with the deposition number of 2258198. Copies of the above data can be obtained free of charge on application at this address: www.ccd.cam.ac.uk.

### 2.4. In Vitro Cytotoxic Activity Screening of Cells with HL-60, A-549, SMMC-7721, MCF-7, SW-480

Compound **1** was screened for in vitro cytotoxic activity in human promyelocytic leukemia cells (HL-60), human lung cancer cells (A-549), human liver cancer cells (SMMC-7721), human breast cancer cells (MCF-7), and human colon cancer cells (SW-480) using the MTT assay by following the below method with cisplatin (DDP) and paclitaxel (Taxol) as positive controls.

1. Cell inoculation: A single cell suspension was obtained from a culture medium (DMEM or RMPI1640) containing 10% fetal bovine serum, and 3000–15,000 cells per well were inoculated onto a 96-well flat-bottomed microtiter plate at a volume of 100 μL per well. The cells were inoculated 12–24 h in advance for culture.
2. Addition of compounds to be tested: Dimethyl sulfoxide (DMSO) solutions containing different concentrations of the tested compound (diluted from 40 μM) were added to a final volume of 200 μL per well, and three replicates were set for each group of treatments.
3. Color development: After incubation at 37 °C for 48 h, the culture solution was discarded for adherent cells, and 20 μL of MTS solution and 100 μL of culture solution were added to each well, respectively; 100 μL of culture supernatant was discarded for suspended cells and 20 μL of MTS solution was added to each well. Incubation was continued for 2~4 h. Then, to measure the light absorption values, 3 blank controls (120 μL of the mixture of MTS solution and culture solution) were set.
4. Colorimetric: A wavelength of 492 nm was selected, and the light absorption value of each well was read by MULTISKAN FC. The results were recorded, and the data were processed and plotted against the compound number as the horizontal coordinate and the cell inhibition rate as the vertical coordinate.
5. Positive control compounds: Two positive compounds, DDP and Taxol, were used in each experiment, and the cell growth curves were plotted with the concentration as the horizontal coordinate and the cell survival rate as the vertical coordinate.

## 3. Results

### 3.1. Structure Identification

Compound **1** was isolated as colorless massive crystals (methanol). Its molecular formula was defined as C_20_H_30_O_3_ by its positive HRESIMS spectrum, which showed a sodium adduct ion peak at *m*/*z* 341.2083 [M + Na]^+^, giving the molecular formula C_20_H_30_O_3_Na calculated as 341.2093, and six degrees of unsaturation. The IR spectrum showed absorption bands at 3434, 1682, and 1631 cm^−1^, which indicate the presence of hydroxyl and carbonyl functionalities. The ^1^H NMR spectrum of compound **1** showed two double-peaked methyl signals at δ_H_ 0.99 (3H, d, J = 7.0 Hz) and 1.23 (3H, d, J = 7.0 Hz), a broad double-peaked methyl signal at δ_H_ 1.53 (3H, br d), two single-peaked methyl signals at δ_H_ 0.81 (3H, s) and 1.08 (3H, s), two oxymethine protons at δ_H_ 4.40 (1H, d, J = 5.5 Hz) and 4.94 (1H, t, J = 5.0 Hz), an exchangeable proton at δ_H_ 1.61 (1H, d, *J* = 5.0 Hz), and a tridisubstituted double bond at δ_H_ 6.05 (1H, s). The ^13^C NMR data (Table 1) showed 20 carbons, including five methyls [δ_C_ 14.9, 15.7, 16.4, 17.8, and 29.1], three methylenes [δ_C_ 26.4, 29.0 and 35.5], seven methines (including one sp^2^ hybrid methine at δ_C_ 114.6, two oxygen-linked methines at δ_C_ 70.9 and 73.1, and four others at δ_C_ 20.0, 22.6, 37.1, and 39.9), and five quaternary carbons (including three sp^2^ hybrid quaternary carbons at δ_C_ 136.7, 145.3, and 177.4, one carbonyl group at δ_C_ 210.1, and one sp^3^ hybrid quaternary carbon at δ_C_ 16.2), which suggests a typical lathyrane-type diterpene with a 5/11/3-membered ring system [16].

The planar structure of compound **1** was determined through careful analysis of its 2D NMR data. The HMBC spectrum of **1** showed correlations of H-1 (δ_H_ 2.44, 2.91) to C-2 (δ_C_ 39.9), C-3 (δ_C_ 210.1), C-4 (δ_C_ 136.7), C-15 (δ_C_ 177.4), and C-16 (δ_C_ 16.4); and H-2 (δ_H_ 2.46) to C-1 (δ_C_ 35.5) and C-3. These data, together with the ^1^H-^1^H COSY cross-peak correlations of H_2_-1/H-2 and H-2/H_3_-16 (δ_H_ 1.23), indicated the existence of a five-membered ring A consisting of C-1, C-2, C-3, C-4, and C-15. The downfield-shifted carbon signals of the α, β-unsaturated ketone (δ_C_ 210.1, 177.4, 136.7) further confirmed the presence of this five-membered ring involving C-3, C-4, and C-15. Furthermore, the ^1^H-^1^H COSY spectrum of **1** gave successive H-7 (δ_H_ 4.40)/H-8 (δ_H_ 2.12, 1.01)/H-9 (δ_H_ 0.93)/H-11 (δ_H_ 0.49)/H-12 (δ_H_ 1.37, 1.02)/H-13 (δ_H_ 1.98)/H3-20 (δ_H_ 0.99) correlations. The HMBC cross-peaks of H-5 (δ_H_ 6.05) to C-6 (δ_C_ 145.3), C-7 (δ_C_ 73.1), C-15, and C-17 (δ_C_ 17.8), as well as H-14 (δ_H_ 4.94) to C-15, linked the above structure fragment to C-5, C-6, and C-15 to afford ring B. The presence of the C ring was determined by correlation of two methine methyls, H_3_-18 (δ_H_ 1.08) and H3-19 (δ_H_ 0.81), with two methines, C-9 (δ_C_ 20.0) and C-11 (δ_C_ 22.6), and one quaternary carbon, C-10 (δ_C_ 16.2), respectively, in the HMBC spectrum. The ^1^H-^1^H COSY correlation of OH-14 (δ_H_ 1.61) with C-14 (δ_C_ 70.9) determined the location of this hydroxyl group, while another hydroxyl group was assigned at C-7 (δ_C_ 73.1) by its chemical shift of δ_C_ 73.1, albeit in the absence of resonances for the hydroxyl group.

**Figure 2:**
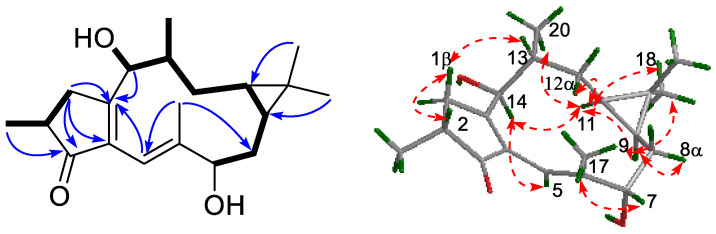
^1^H-^1^H COSY (—), key HMBC (⟶), and ROESY (⟷) correlations of compound **1**.

**Figure 3:**
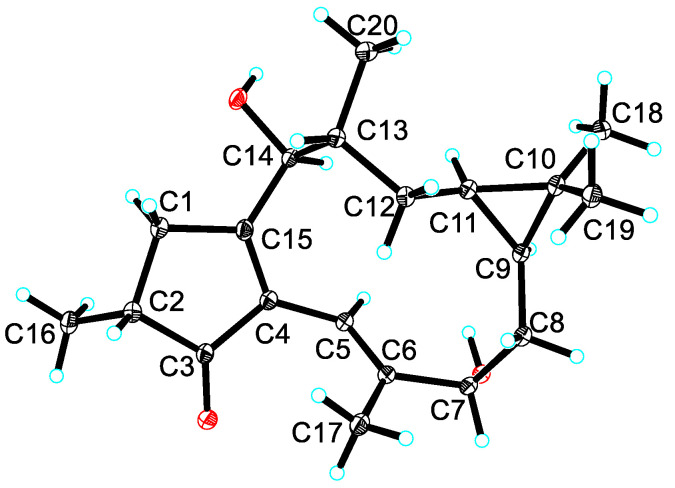
ORTEP drawing of compound **1**.

The relative conformation of compound **1** was determined by the ROESY experiments, which showed the following correlation signals (Figure 2): H-14 (δ_H_ 4.94)/H-11 (δ_H_ 0.49), H-11/H-9 (δ_H_ 0.93), H-11/H_3_-20 (δ_H_ 0.99), H-14/H-5 (δ_H_ 6.05), H-11/H_3_-18 (δ_H_ 1.08), and H-9 (δ_H_ 0.93)/H_3_-18. These data suggested that H-14, H3-20, H-9, H-11, and H_3_-18 are cofacial and assigned as α-oriented and determined the β-configurations of H-13 and OH-14 simultaneously. The ROESY cross-peaks of H-13 (δ_H_ 1.98)/H-1β (δ_H_ 2.91) and H-1β/H-2 (δ_H_ 2.46) determined H-2 as a β-configuration and H_3_-16 as an α-configuration. However, the H-7 (δ_H_ 4.40)/H_3_-17 (δ_H_ 1.53) cross-peaks were insufficient to determine their conformations. Fortunately, the crystals of compound **1** were obtained, which confirmed the correctness of the relative conformation determined by the ROESY spectrum described above and assigned H-7 as a β-configuration and OH-7 as an α-configuration. Interestingly, although the planar structures of compound **1**, kansuingols A and B, are quite similar, there are many differences in their relative conformations, that is, methyl-16, methyl-20, and hydroxyl-7 of compound **1** are α-oriented, while the corresponding groups of kansuingols A and B are β-oriented [17]. Finally, the Flack parameter value of the crystal structure of compound **1** was 0.03(9), which led to the determination of its absolute configuration, as shown in Figure 3.

Furthermore, based on the comparison of the NMR data with those reported in the literature, the known compounds euphopepluanones D (**2**) [18], 5-O-benzoyl-20-deoxymgenol (**3**) [19], 3-O-benzoyl-20-deoxymgenol (**4**) [19], ingenol-20-angelate **(5**) [20], helioscopinolide A (**6**) [21], 7α-hydroxy-8α,14-dihydro jolkinolide E (**7**) [22], 5,6-epoxy-3-hydroxy-7-megastigmen-9-one (8) [23], PBI 344 (**9**) [24], kaempferol (**10**) [25], and naringenin (11) [26] were identified.

### 3.2. Cytotoxicity

The isolated compounds were screened and evaluated for their cytotoxicities against five human cancer cell lines (namely, human promyelocytic Leukemia cells HL-60, human lung cancer cells A-549, human liver cancer cells SMMC-7721, human breast cancer cells MCF-7, and human colon cancer cells SW480), with DDP and Taxol as two positive controls. Only compound **4** showed moderated cytotoxic activity against HL-60, A-549, SMMC-7721, MCF-7, and SW480 cell lines, with IC_50_ values of 10.5 ± 0.18, 21.47 ± 0.17, 18.36 ± 1.17, 18.82 ± 0.84, and 16.25 ± 0.71 μM, respectively; while DDP has IC_50_ values of 10.28 ± 0.21, 29.70 ± 0.72, 13.66 ± 0.76, 27.28 ± 1.23, and 24.13 ± 1.09; Taxol has IC_50_ values of <0.008, 0.127 ± 0.007, <0.008, <0.008, and <0.008, respectively. The other compounds were inactive at a concentration of 40 μM.

## 4. Discussion

*Euphorbia* plants are known for their irritant milky latex [27] and characteristic and chemically diverse diterpenoids. Among the macrocyclic and polycyclic diterpenoids from *Euphorbia*, jatrophane types are the most common [28,29,30,31]. To date, the diterpenoids reported from *E. stracheyi* have included *ent*-kaurane, *ent*-abietane, *ent*-atisane, *ent*-isopimarane, labdane, tigliane, lathyrane, and ingenane-type diterpenoids [10,11,12,13,14], but no jatrophanes were isolated from the species. In the present study, besides the new compound euphostrachenol A, one lathyrane-type, three ingenane-type, and two abietane-type diterpenoids were isolated from *E. stracheyi,* all of which are known compounds and were discovered from other species of *Euphorbia,* suggesting a similar diterpenoid profile of these species. Importantly, our phytochemical investigation of *E. stracheyi* reported here also yielded no jatrophane-type diterpenoids, in accordance with the previous results. This type of diterpenoid is found exclusively in the *Euphorbiaceae* family, and it has a 5/12 bicyclic pentadecane skeleton, in which the absence of a cyclopropane ring is the most obvious structural feature that differentiates it from other macrocyclic and polycyclic diterpenoids from *Euphorbia,* such as tigliane, lathyrane, and ingenane types. The absence of jatrophanes in the species may be related to the special environment in which *E. stracheyi* lives, namely, the high-altitude region, which is often associated with high loads of UV radiation and low temperatures. Jatrophane-type diterpenoids exhibit many different activities, including antitumor, antiviral, antifungal, and anti-inflammatory effects [2,3]. Many of them showed promising P-glycoprotein, a membrane protein that pumps anticancer drugs out of cells, exhibits inhibitor activity, and could be developed as a new drug to reverse multidrug resistance [2,3]. Thus, the pharmacological value of *E. stracheyi* may be influenced. Furthermore, the lack of jatrophanes implied that its biosynthetic pathway may be lost in the plant of *E. stracheyi*. Currently, jatrophanes were suggested to be derived from lathyrane-type diterpenoids by combined transcriptomic, genomic, and metabolomic investigation of *E. peplus*, a species harboring many jatrophane-type diterpenoids [32]. Thus, comparative genomic and transcriptomic analysis between these two species could provide useful clues for uncovering the biosynthesis of jatrophane-type diterpenoids.

Notably, ionones are rarely isolated from the species of *Euphorbia* [2]; however, they were reported constituents of *E. stracheyi* [8,12]. We also reported two ionones from *E. stracheyi*, in agreement with previous studies. Ionones are degraded from carotenoids that play an important role in helping plants adapt to high UV light and low temperatures. During photosynthesis, carotenoids are known to protect chlorophylls and bacteriochlorophylls from sensitizing deleterious photodestructive reactions [33]. The detection of ionones in *E. stracheyi* in the present and previous studies strongly suggested that this type of secondary metabolite should be the result of high-altitude adaptation of the species. Further work should be undertaken to reveal whether other *Euphorbia* plants adapted to the harsh environments of Tibetan peaches also harbor ionones.

Notably, the planar structure of compound **1** shows high similarity to those of kansuingols A and B, except for a β–D-glucose group and a hydroxyl group present at C-15 and C-19 of kansuingol A, as well as a β–D-glucose group at C-19 of kansuingol B, respectively [17]. Notably, the relative conformations of these three compounds are also different for methyl-16, methyl-20, and hydroxyl-7. While the relative conformations of compound **1** were supported by X-ray diffraction analysis, those of kansuingols A and B were not. After checking the key ROESY correlations that determined the relative conformation assignment of methyl-16, methyl-20, and hydroxyl-7 in kansuingols A and B, we found that there are no ROESY correlations to support the claimed β-orientation of methyl-16 in both compounds. Therefore, the structures of kansuingols A and B remain to be determined.

## Figures and Tables

**Table 1:** NMR data of compound **1** (500 MHz for 1H/and 125 MHz for ^13^C, CDCl_3_).

Carbon Position	δ_H_	δ_C_	Carbon Position	δ_H_	δ_C_
1α	2.44 (1H, m)	35.5	11	0.49 (1H, ddd, *J* = 12.5, 7.5, 5.0 Hz)	22.6
1β	2.91 (1H, m)		12α	1.37 (1H, ddd, *J* = 12.5, 7.5, 5.0 Hz)	26.4
2	2.46 (1H, m)	39.9	12β	1.02 (1H, m)	
3		210.1	13	1.98 (1H, m)	37.1
4		136.7	14	4.94 (1H, t, *J* = 5.0 Hz)	70.9
5	6.05 (1H, s)	114.6	15		177.4
6		145.3	16	1.23 (3H, d, *J* = 7.0 Hz)	16.4
7	4.40 (1H, d, *J* = 5.5 Hz)	73.1	17	1.53 (3H, br d)	17.8
8α	2.12 (1H, ddd, *J* = 14.5, 5.5, 1.0 Hz)	29.0	18	1.08 (3H, s)	29.1
8β	1.01 (1H, m)		19	0.81 (3H, s)	15.7
9	0.93 (1H, m)	20.0	20	0.99 (3H, d, *J* = 7.0 Hz)	14.9
10		16.2	14-OH	1.61 (1H, d, *J* = 5.0 Hz)	

## Data Availability

The data presented in this study are available in the main article and the Supplementary Materials.

## Supplementary Materials

The following supporting information can be downloaded at: https://www.mdpi.com/article/10.3390/metabo13070852/s1, Figures S1–S31: Spectrums of compounds **1**–**11**; Table S1: Crystal data and structure refinement for compound **1**. Table S2: Cytotoxicity data of compounds **1**–**11** at the concentration of 40 μM.
