# Supplementary material for: Chemical Constituents of Euphorbia stracheyi Boiss (Euphorbiaceae)
Source: Metabolites. 2023 Jul 15;13(7):852. doi: 10.3390/metabo13070852 (PMC10384431; doi:10.3390/metabo13070852)
Supplement: Supplementary file 1 [file metabolites-13-00852-s001.zip › metabolites-2415199-supplementary.pdf]

Supporting Information

Chemical constituents of *Euphorbia stracheyi* Boiss (Euphorbiaceae)

Hui Zhu [1,2], Xiangxiang Ren [1,2], Tao Su [1] and Lei Yang [2,*]

Co-Innovation Center for Sustainable Forestry in Southern China，College of Biology and the Enviroment，Nanjing Forestry University，Nanjing 210037，China, zh8201711509@njfu.edu.cn, renxiangxiang@njfu.edu.cn , sutao@njfu.edu.cn
Shanghai Key Laboratory of Plant Functional Genomics and Resources, Shanghai Chenshan Botanical Garden, Shanghai, 201602, China
* Correspondence: leiyang@cemps.ac.cn; Tel.: 86-21-37792288

Contents of Supporting Information

Figure S1.^{1}H NMR ($CDCl_3$) spectrum of compound **1** 3
Figure S2.^{13}C NMR ($CDCl_3$) spectrum of compound **1** 3
Figure S3. DEPT135 NMR ($CDCl_3$) spectrum of compound **1** 4
Figure S4. DEPT90 NMR ($CDCl_3$) spectrum of compound **1** 4
Figure S5. HSQC ($CDCl_3$) spectrum of compound **1** 5
Figure S6. ^{1}H-^{1}H COSY ($CDCl_3$) spectrum of compound **1**...5
Figure S7. HMBC ($CDCl_3$) spectrum of compound **1** 6
Figure S8. ROESY ($CDCl_3$) spectrum of compound **1**..6
Figure S9. HRESIMS spectrum of compound **1**..7
Figure S10. IR spectrum of compound **1** 7
Figure S11. UV spectrum of compound **1**...8
Figure S12.^{1}H NMR ($CDCl_3$) spectrum of compound **2** 8
Figure S13.^{13}C NMR ($CDCl_3$) spectrum of compound **2** 9
Figure S14.^{1}H NMR ($CDCl_3$) spectrum of compound **3** 9
Figure S15.^{13}C NMR ($CDCl_3$) spectrum of compound **3** 10
Figure S16.^{1}H NMR ($CDCl_3$) spectrum of compound **4** 10
Figure S17.^{13}C NMR ($CDCl_3$) spectrum of compound **4** 11
Figure S18.^{1}H NMR ($CDCl_3$) spectrum of compound **5** 11
Figure S19.^{13}C NMR ($CDCl_3$) spectrum of compound **5** 12
Figure S20.^{1}H NMR ($CDCl_3$) spectrum of compound **6** 12
Figure S21.^{13}C NMR ($CDCl_3$) spectrum of compound **6** 13
Figure S22.^{1}H NMR ($CDCl_3$) spectrum of compound **7** 13
Figure S23.^{13}C NMR ($CDCl_3$) spectrum of compound **7** 14
Figure S24.^{1}H NMR ($CDCl_3$) spectrum of compound **8** 14
Figure S25.^{13}C NMR ($CDCl_3$) spectrum of compound **8** 15
Figure S26.^{1}H NMR ($CDCl_3$) spectrum of compound **9** 15
Figure S27.^{13}C NMR ($CDCl_3$) spectrum of compound **9** 16
Figure S28.^{1}H NMR (CD_3OD) spectrum of compound **10** 16
Figure S29.^{13}C NMR (CD_3OD) spectrum of compound **10** 17
Figure S30.^{1}H NMR (CD_3OD) spectrum of compound **11** 17
Figure S31.^{13}C NMR (CD_3OD) spectrum of compound **11** 18
Table S1. Crystal data and structure refinement for compound **1**.................................19
Table S2. Cytotoxicity data of compounds **1-11** at the concentration of 40 μM 20

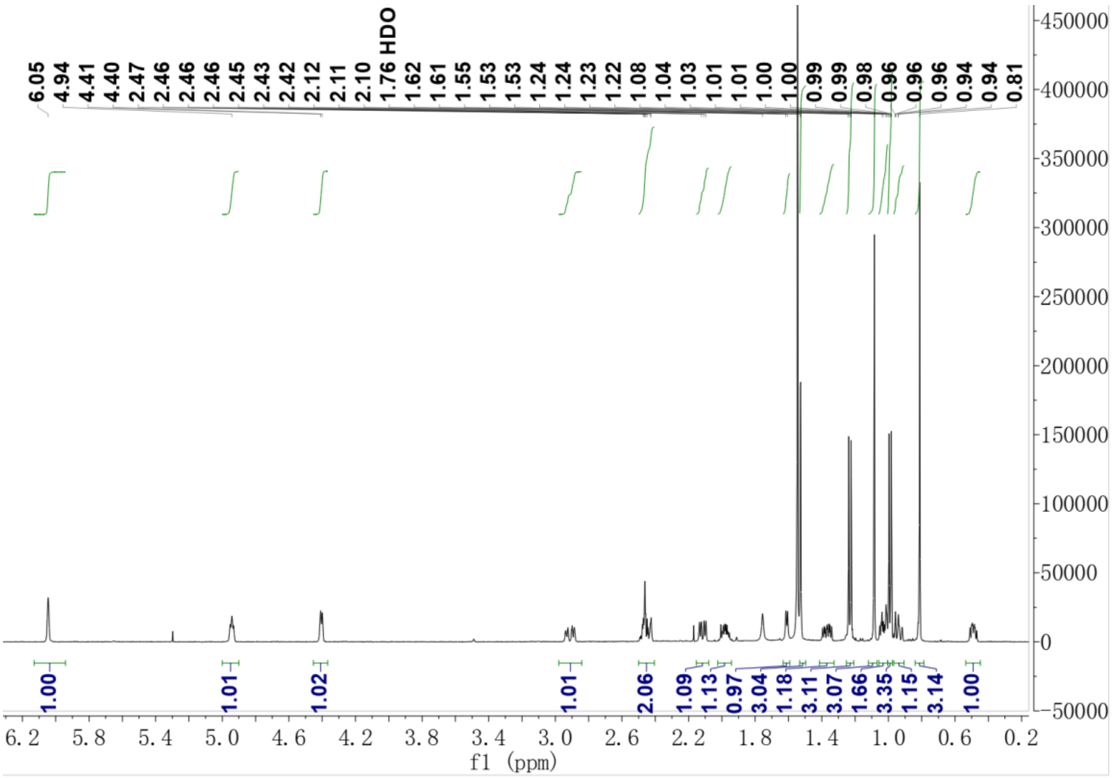

Figure S1. ^{1}H NMR ($CDCl_3$) spectrum of compound **1**

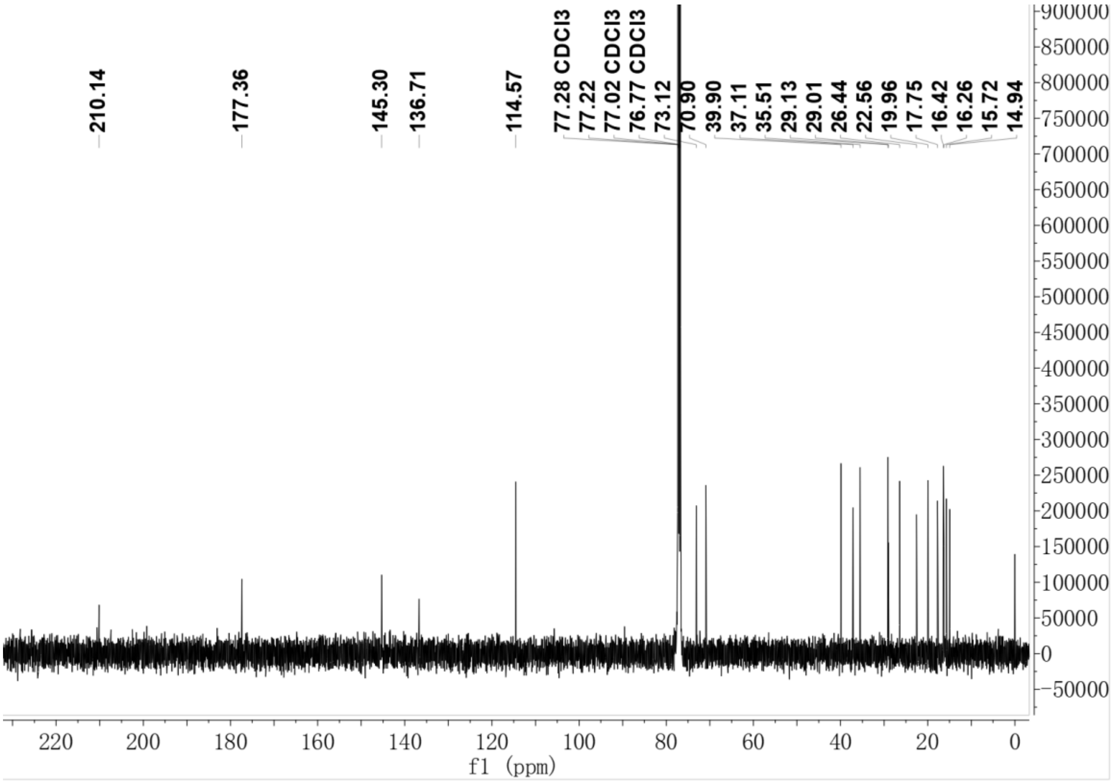

Figure S2. ^{13}C NMR ($CDCl_3$) spectrum of compound **1**

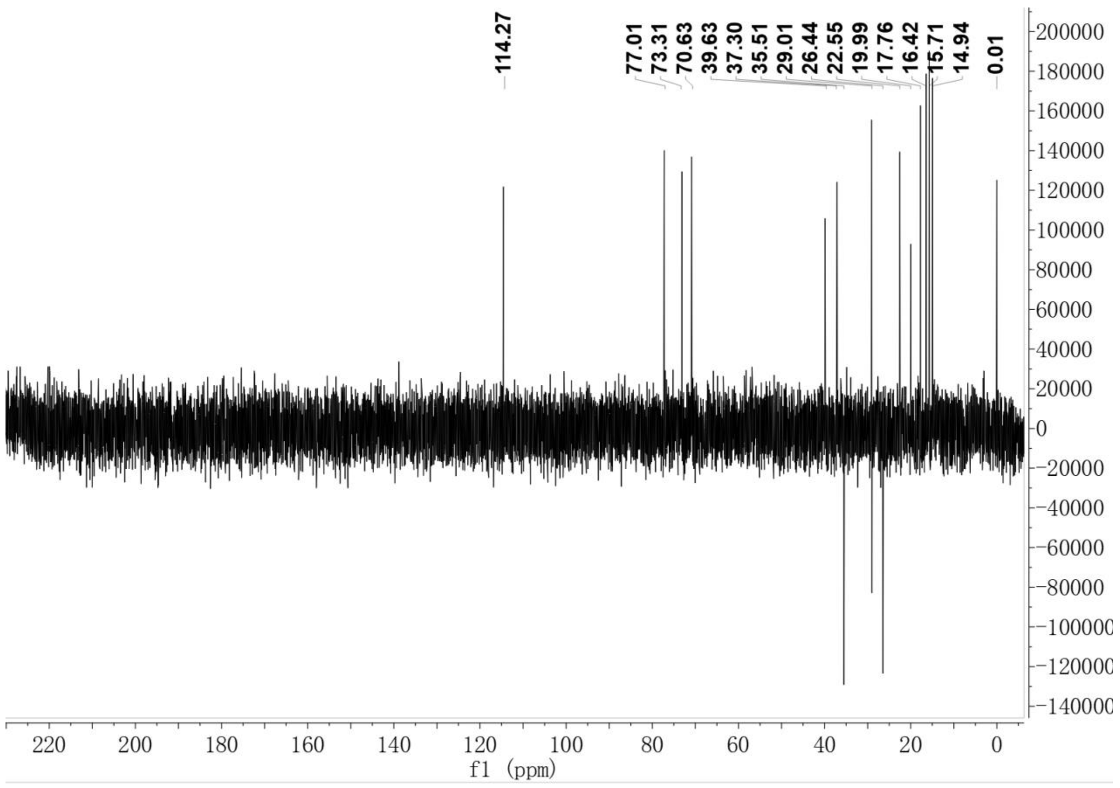

Figure S3. DEPT135 NMR ($CDCl_3$) spectrum of compound **1**

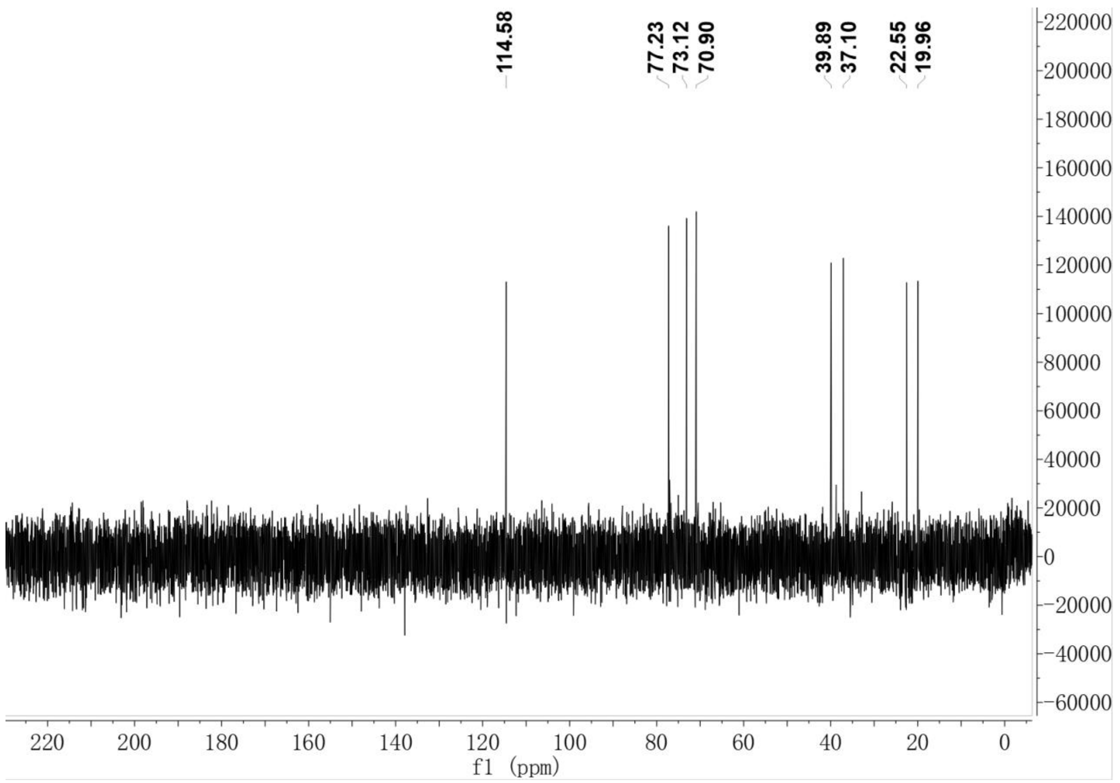

Figure S4. DEPT90 NMR ($CDCl_3$) spectrum of compound **1**

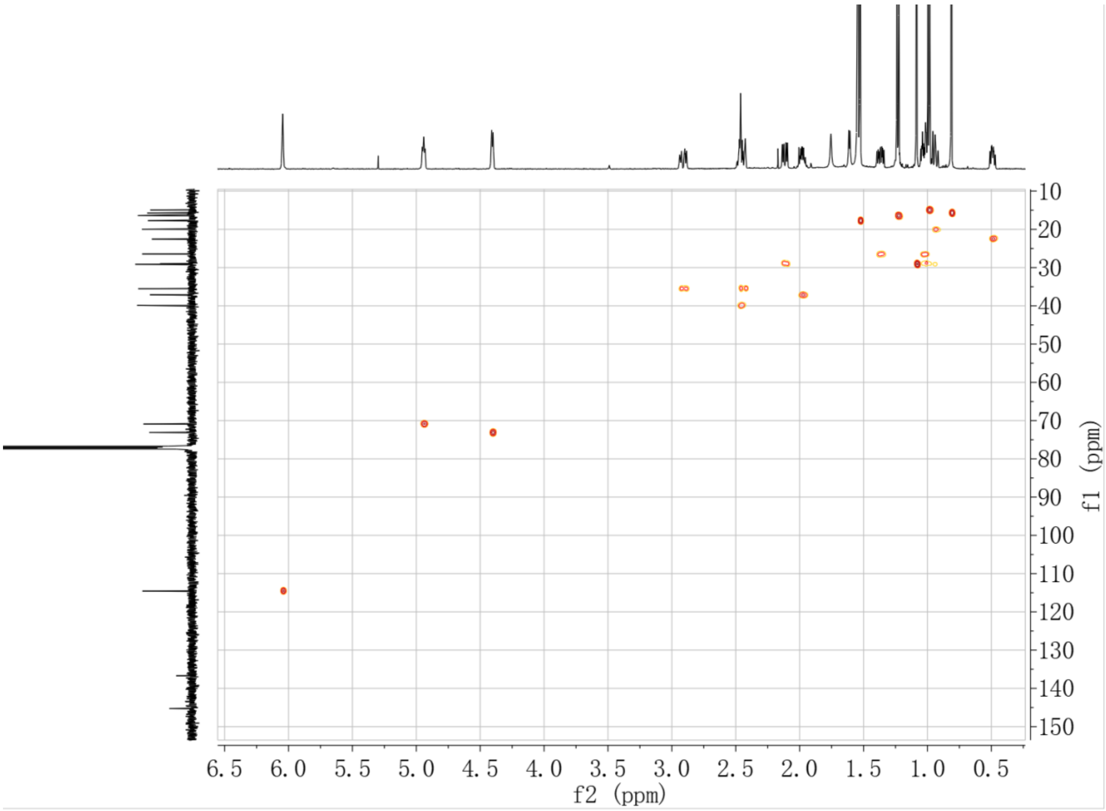

Figure S5. HSQC ($CDCl_3$) spectrum of compound **1**

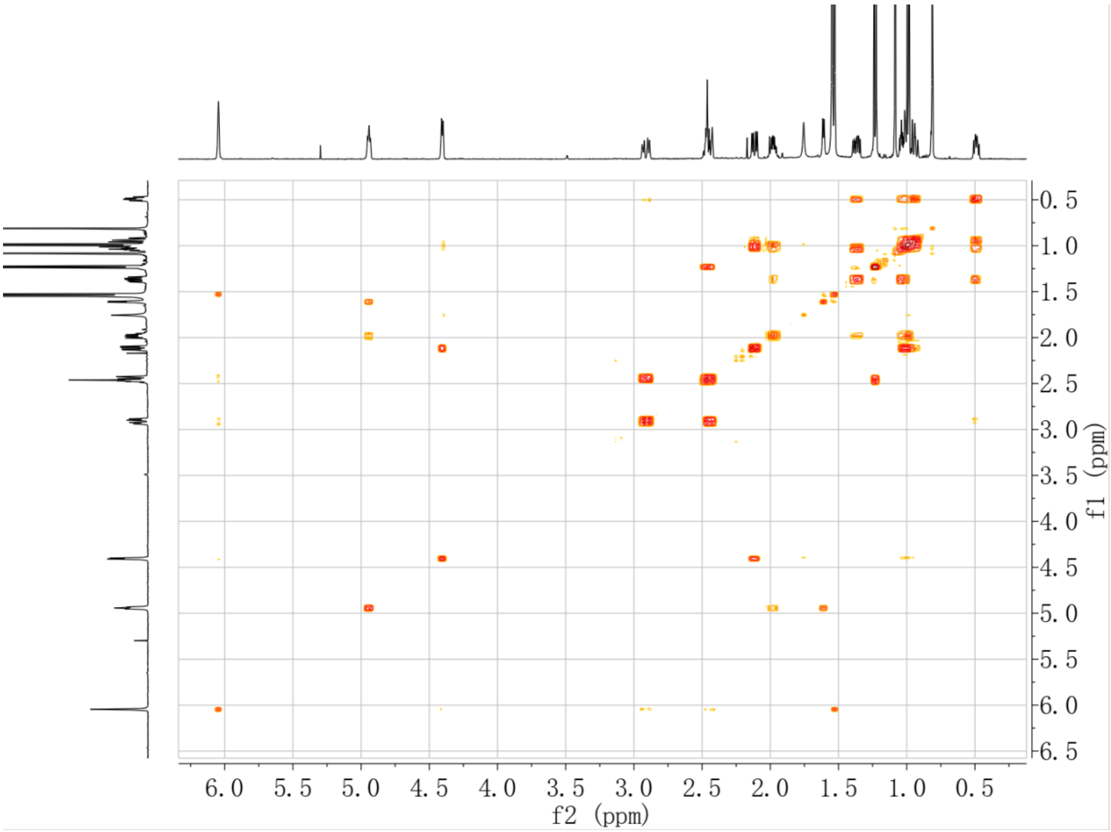

Figure S6. ^1H-^1H COSY ($CDCl_3$) spectrum of compound **1**

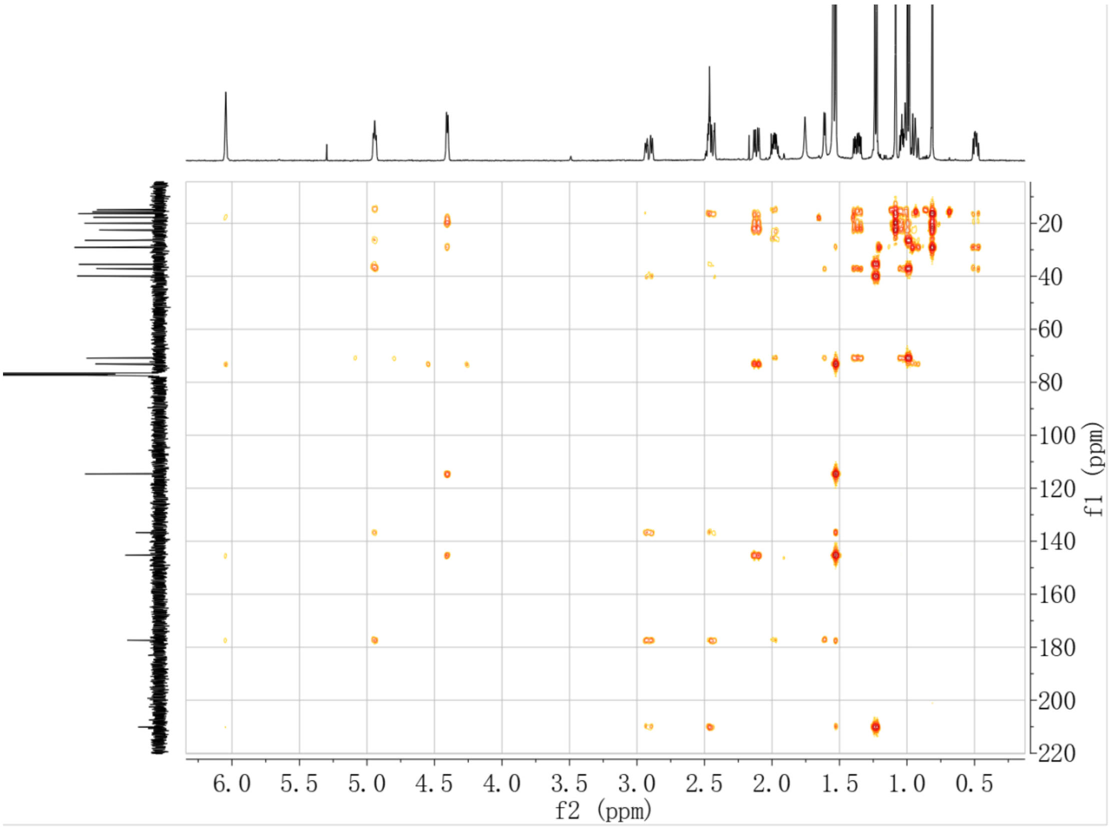

Figure S7. HMBC ($CDCl_3$) spectrum of compound **1**

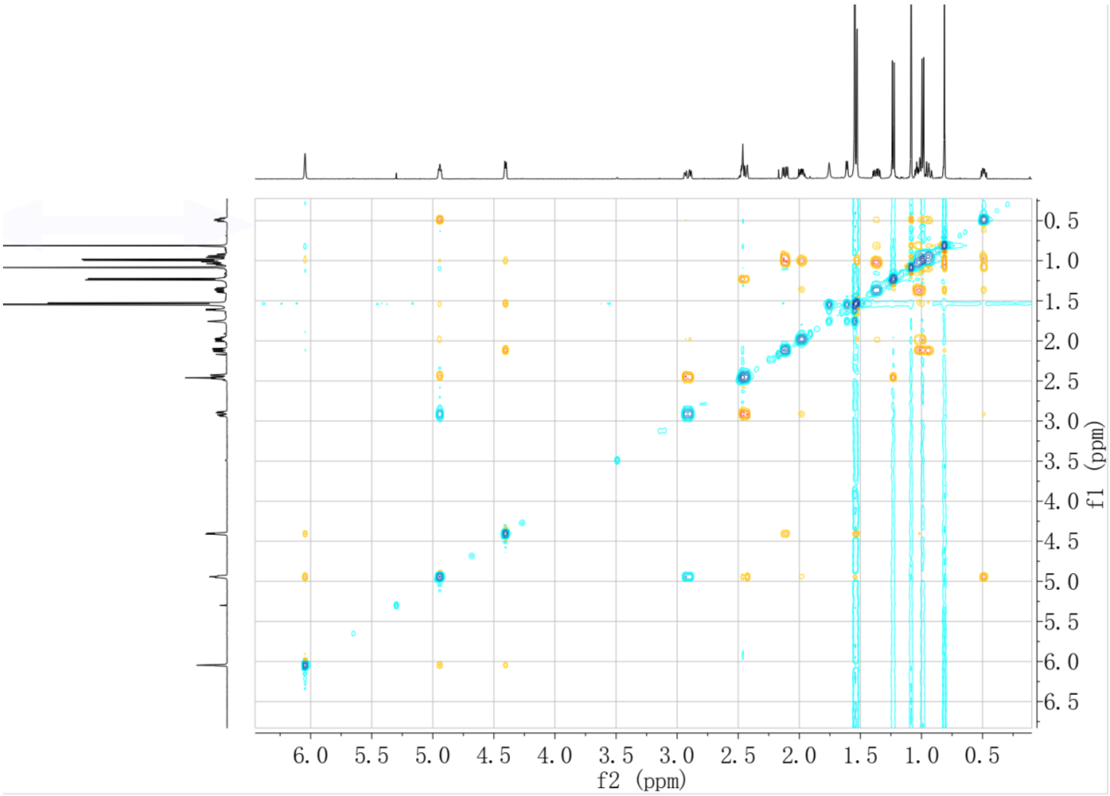

Figure S8. ROESY ($CDCl_3$) spectrum of compound **1**

User Spectra

Fragmentor Voltage	Collision Energy	Ionization Mode
170	0	ESI

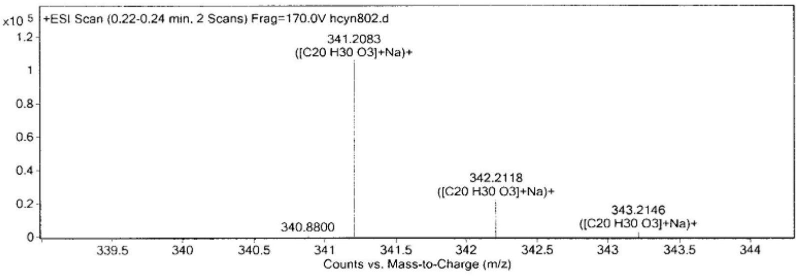

Peak List

m/z	z	Abund	Formula	Ion
128.953		29637.79		
150.112	1	15434.45		
172.0941	1	85035.27		
319.2264	1	22794.99		
341.2083	1	105891.84	C20 H30 O3	(M+Na)+
342.2118	1	22476.57	C20 H30 O3	(M+Na)+
357.182	1	28981.82		
363.19	1	12112.17		
659.4278	1	65302.52		
660.4309	1	28264.04		

Formula Calculator Element Limits

Element	Min	Max
C	3	60
H	0	120
O	0	30

Formula Calculator Results

Formula	CalculatedMass	CalculatedMz	Mz	Diff. (mDa)	Diff. (ppm)	DBE
C20 H30 O3	318.2195	341.2087	341.2083	0.40	1.17	6.0000

--- End Of Report ---

Figure S9. HRESIMS spectrum of compound **1**

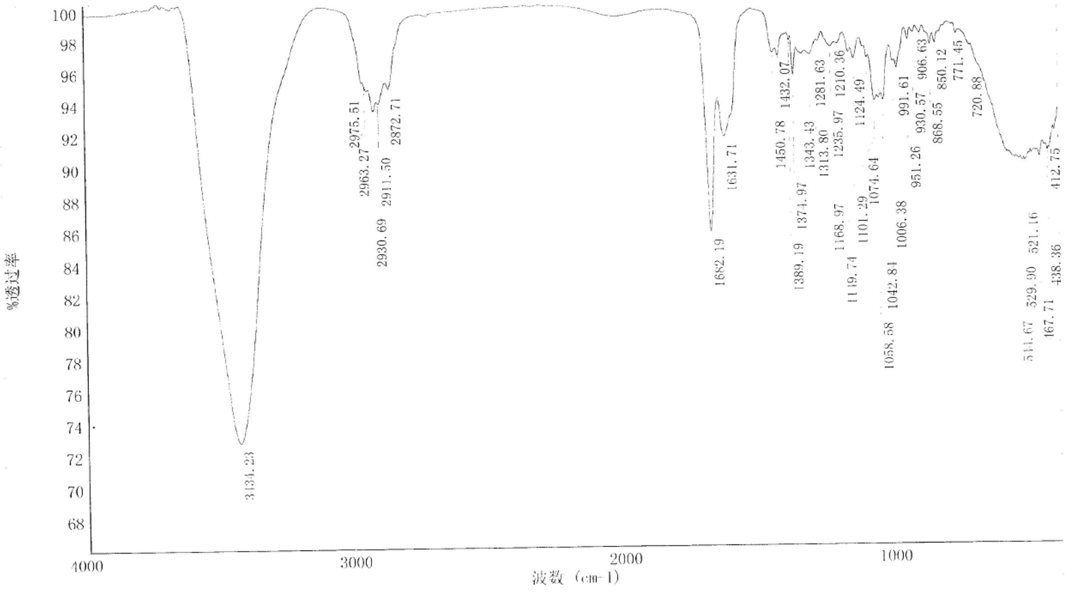

Figure S10. IR spectrum of compound **1**

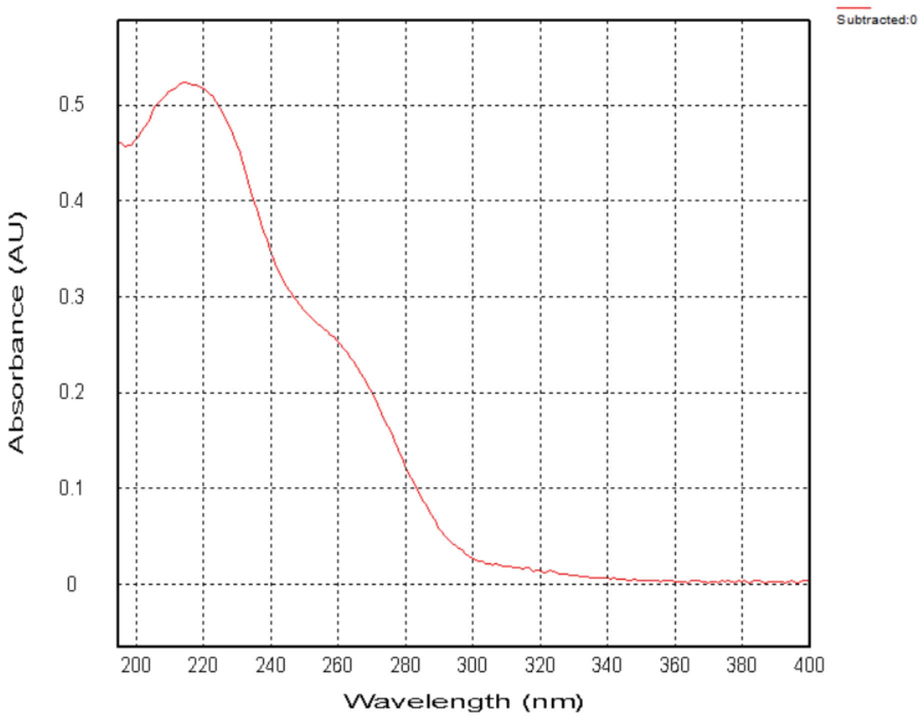

Figure S11. UV spectrum of compound **1**

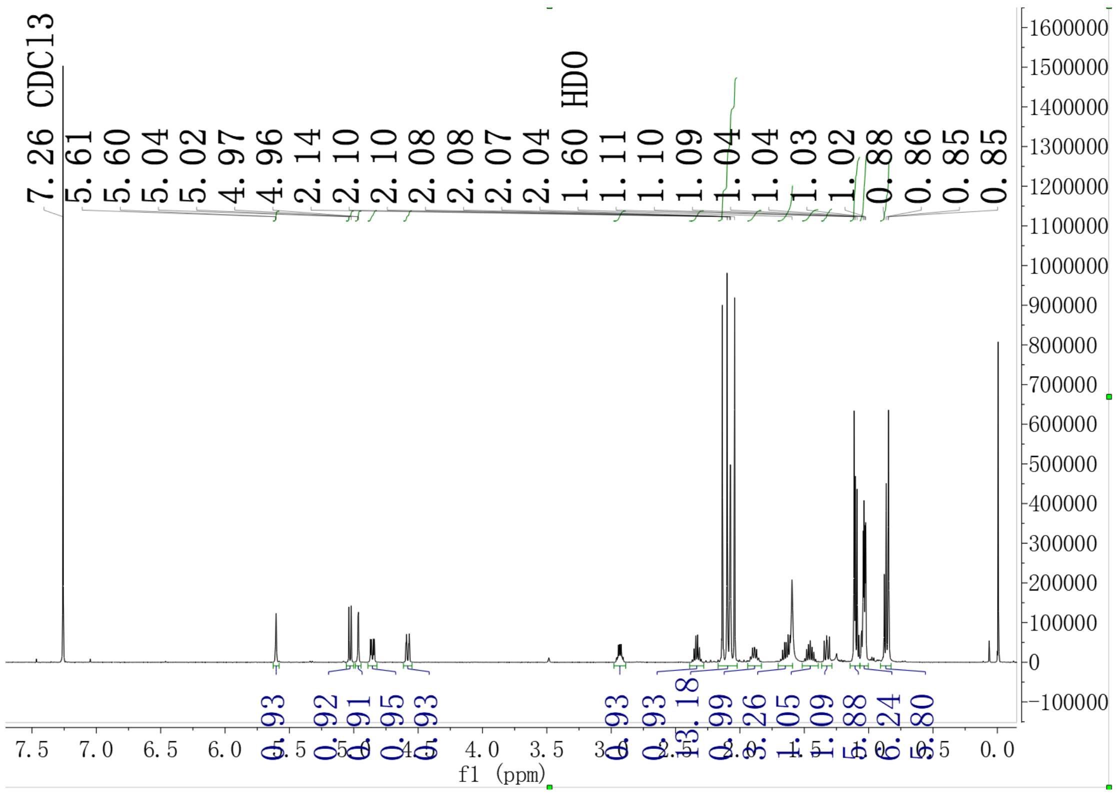

Figure S12. ^{1}H NMR (CDCl3) spectrum of compound **2**

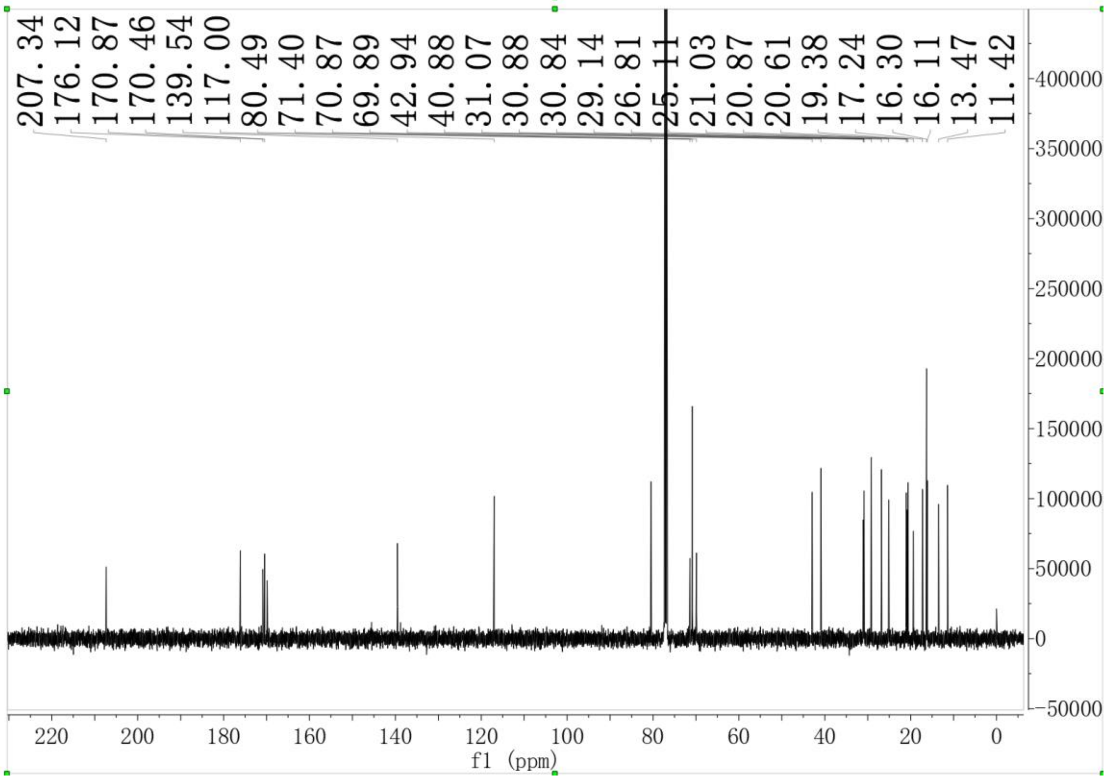

Figure S13.^{13}C NMR ($CDCl_3$) spectrum of compound **2**

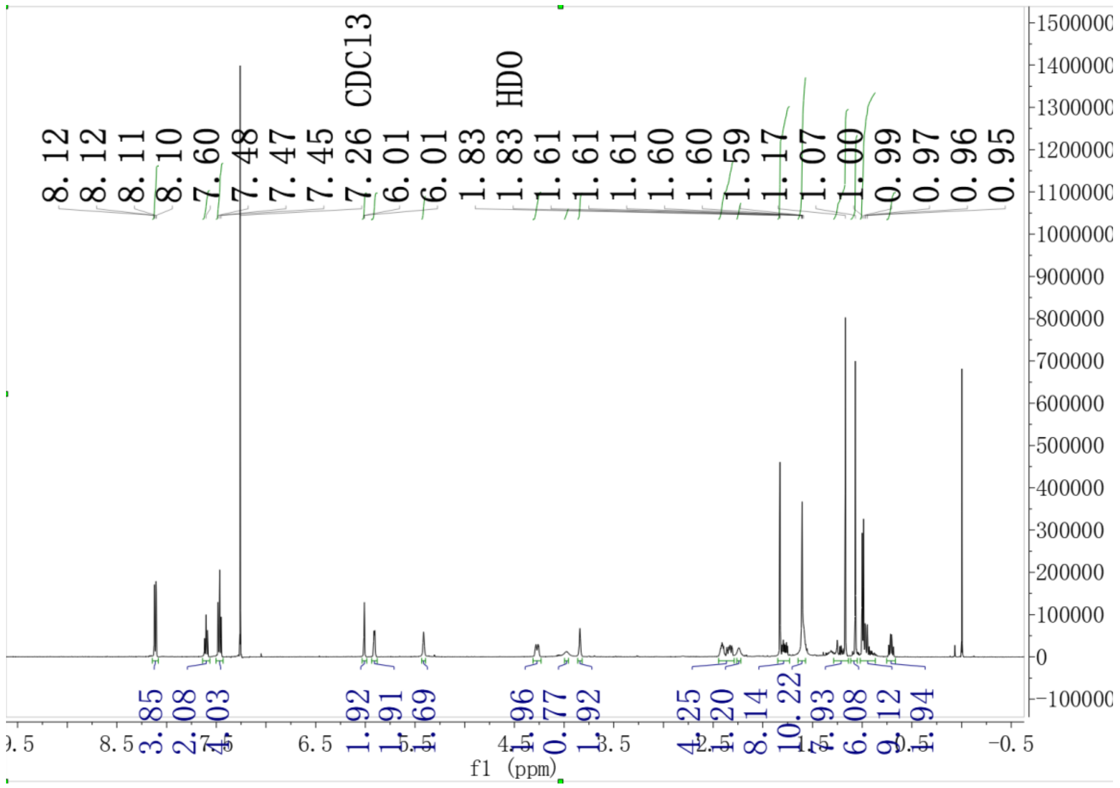

Figure S14. ^{1}H NMR ($CDCl_3$) spectrum of compound **3**

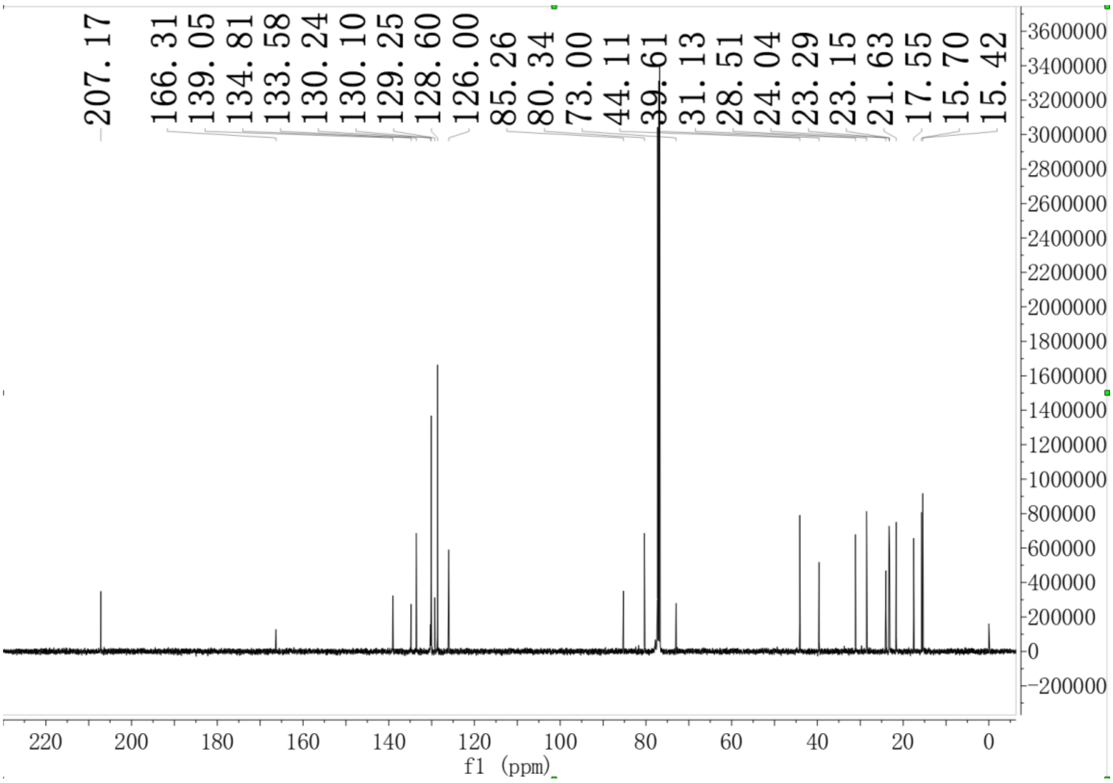

Figure S15.^{13}C NMR ($CDCl_3$) spectrum of compound **3**

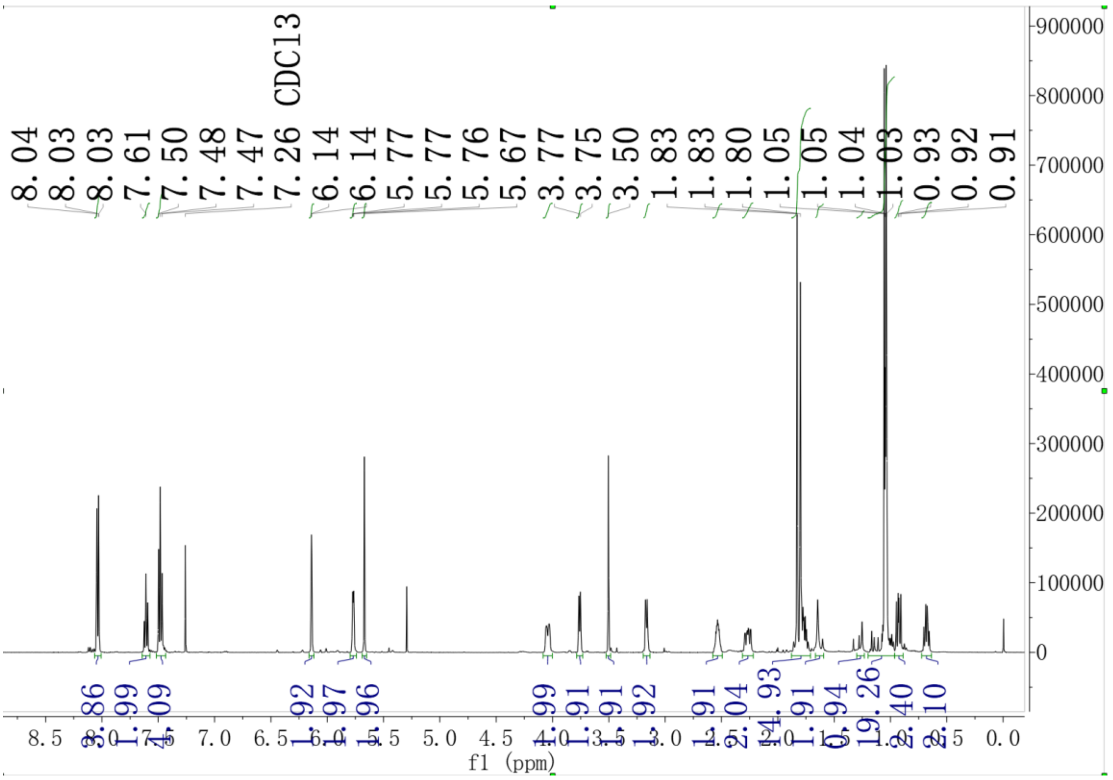

Figure S16. ^{1}H NMR ($CDCl_3$) spectrum of compound **4**

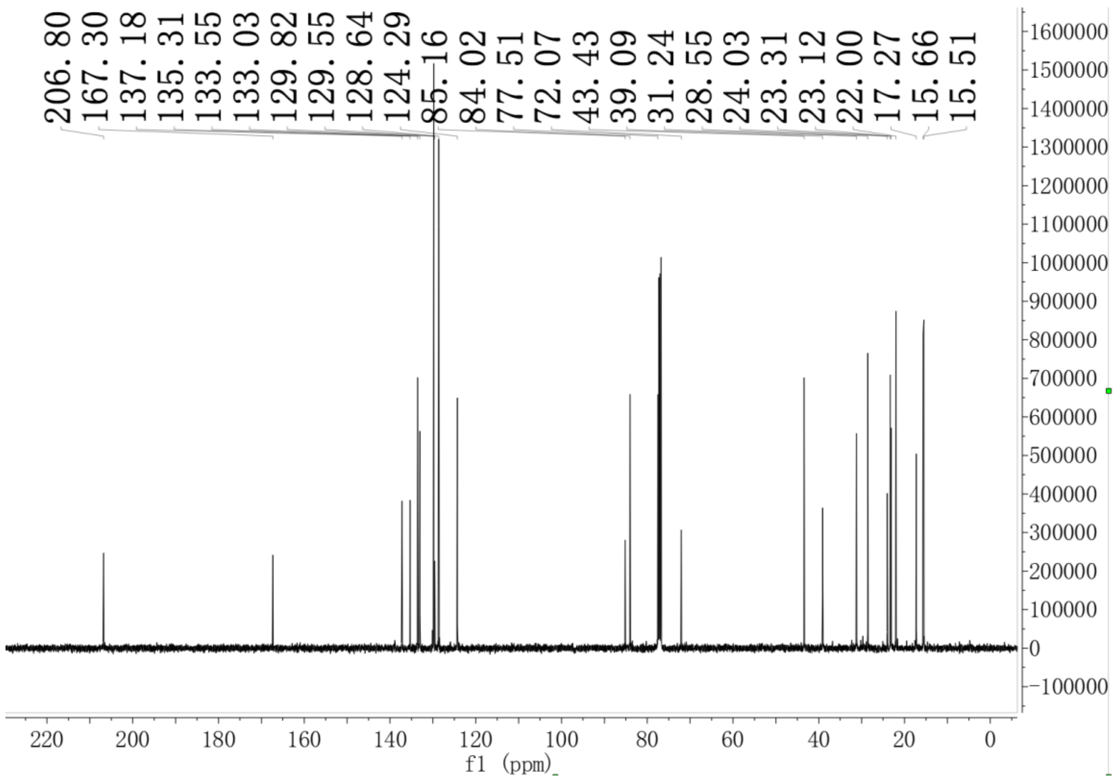

Figure S17. ^{13}C NMR (CDCl3) spectrum of compound **4**

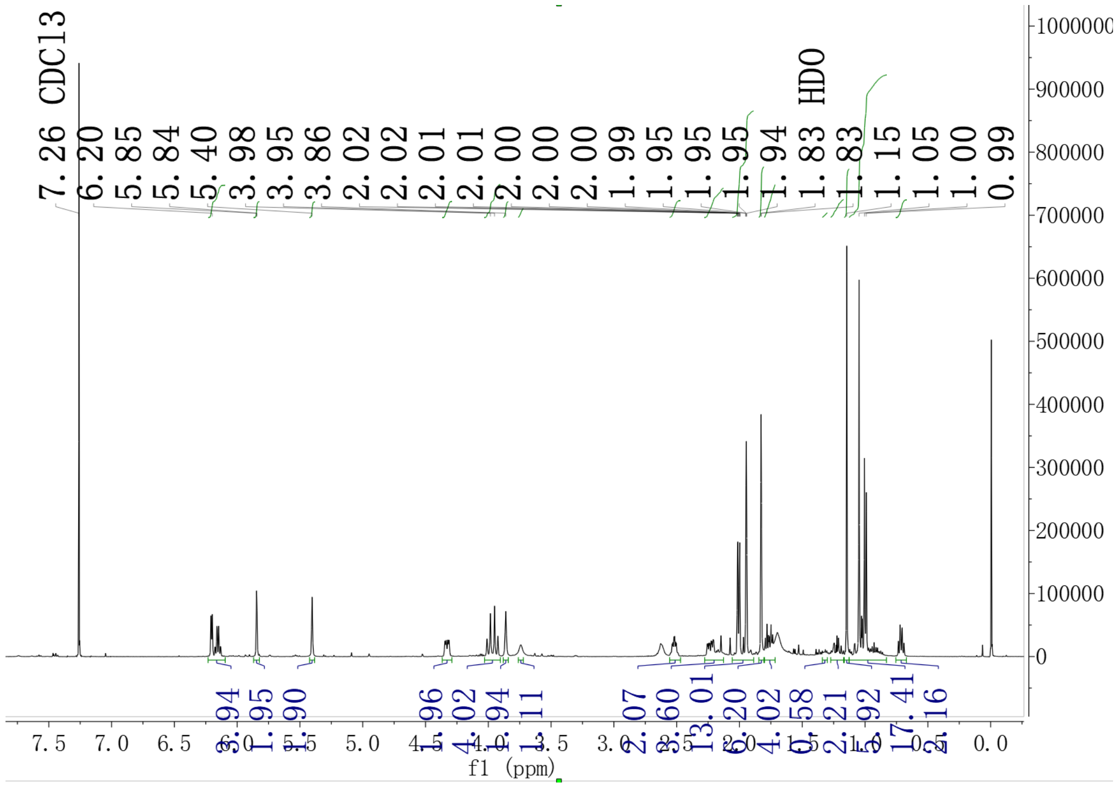

Figure S18. ^{1}H NMR (CDCl3) spectrum of compound **5**

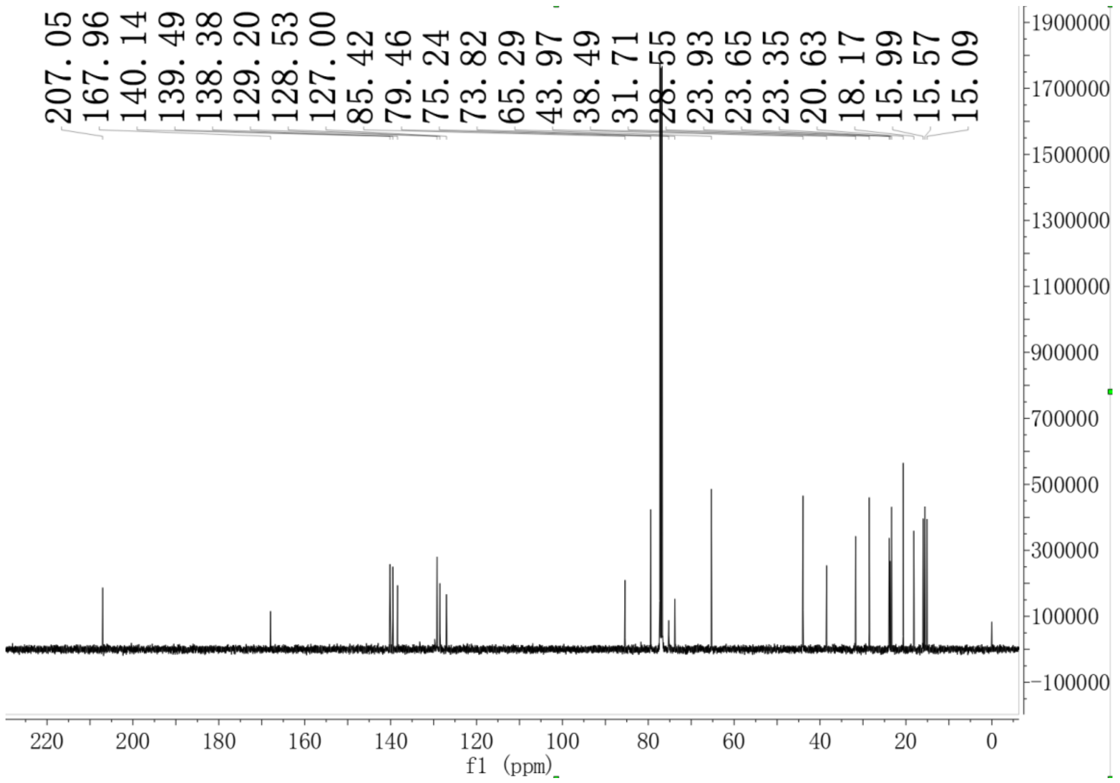

Figure S19.^{13}C NMR ($CDCl_3$) spectrum of compound **5**

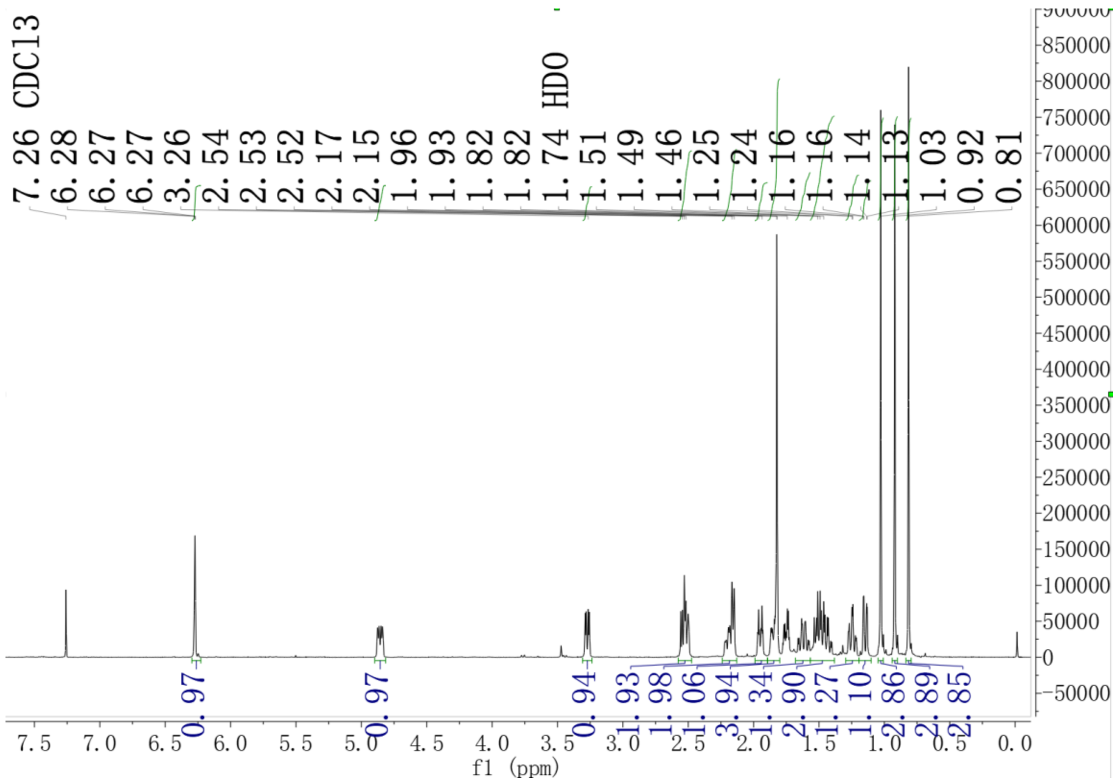

Figure S20. ^{1}H NMR ($CDCl_3$) spectrum of compound **6**

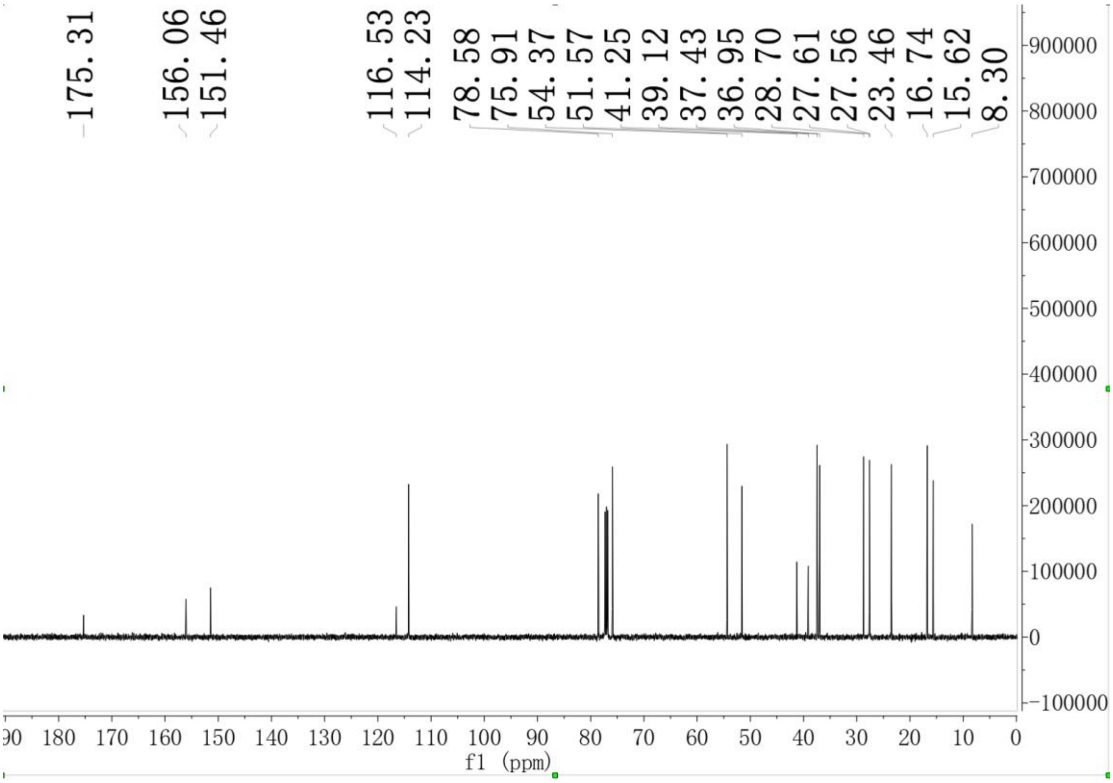

Figure S21. ^{13}C NMR ($CDCl_3$) spectrum of compound **6**

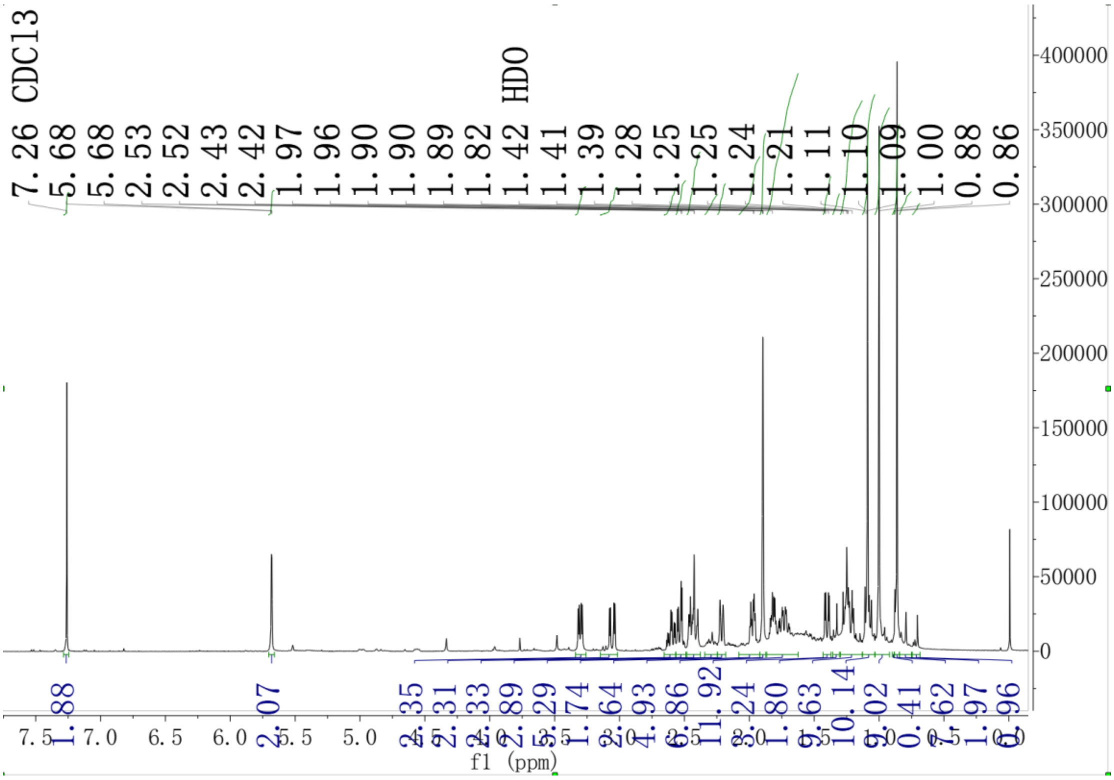

Figure S22. ^{1}H NMR ($CDCl_3$) spectrum of compound **7**

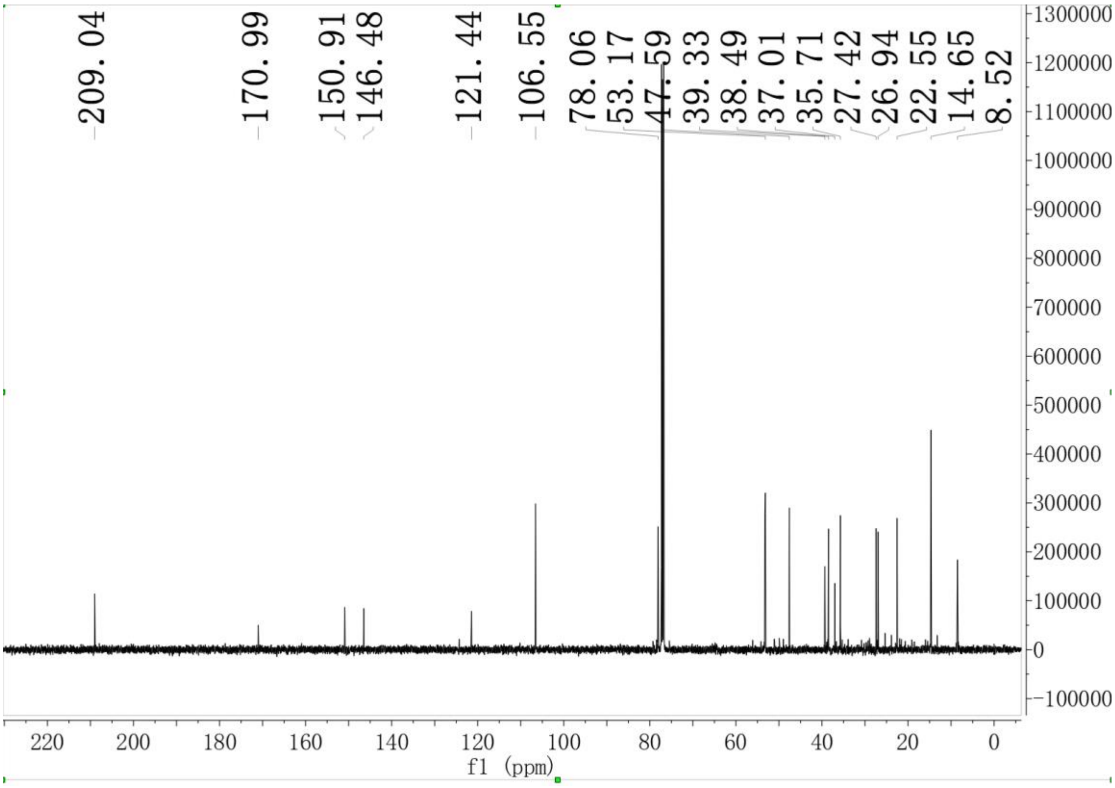

Figure S23.^{13}C NMR (CDCl3) spectrum of compound **7**

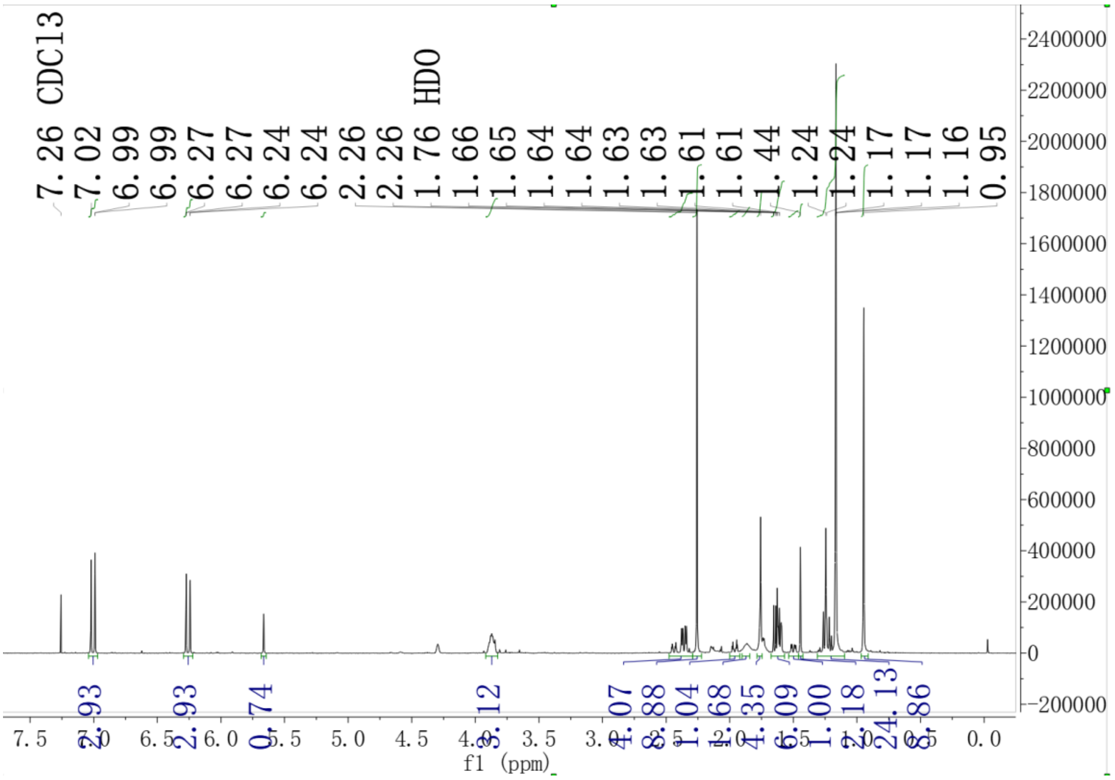

Figure S24. ^{1}H NMR (CDCl3) spectrum of compound **8**

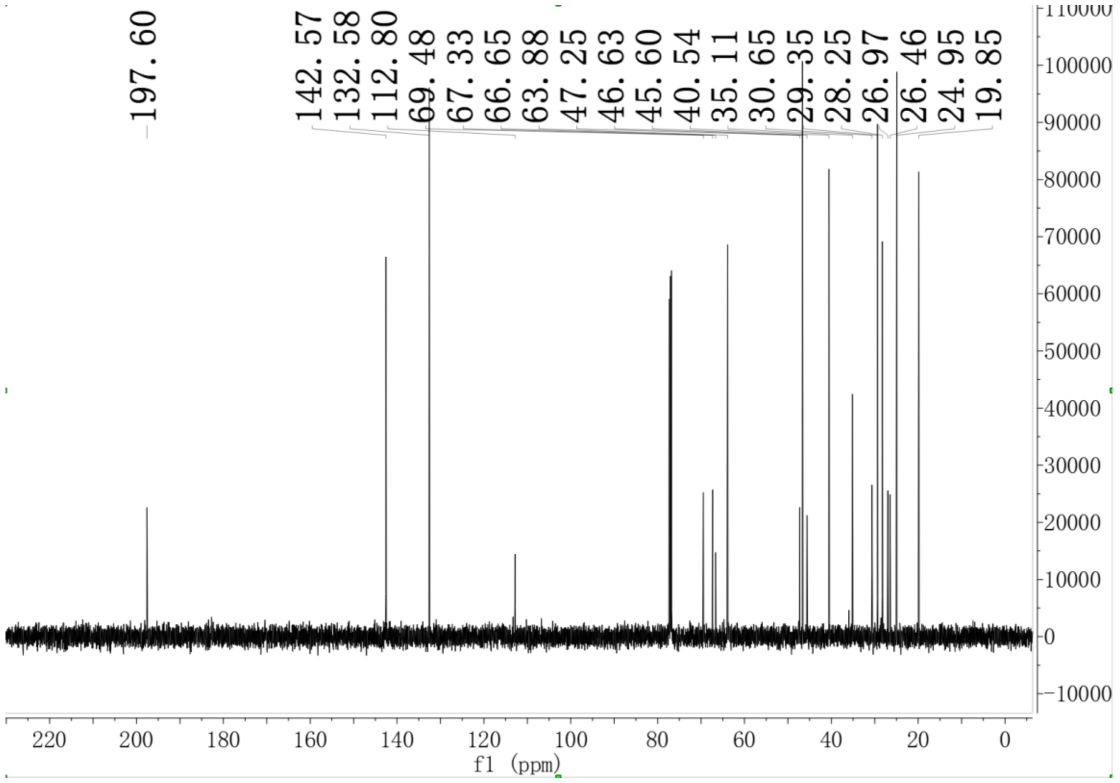

Figure S25.^{13}C NMR ($CDCl_3$) spectrum of compound **8**

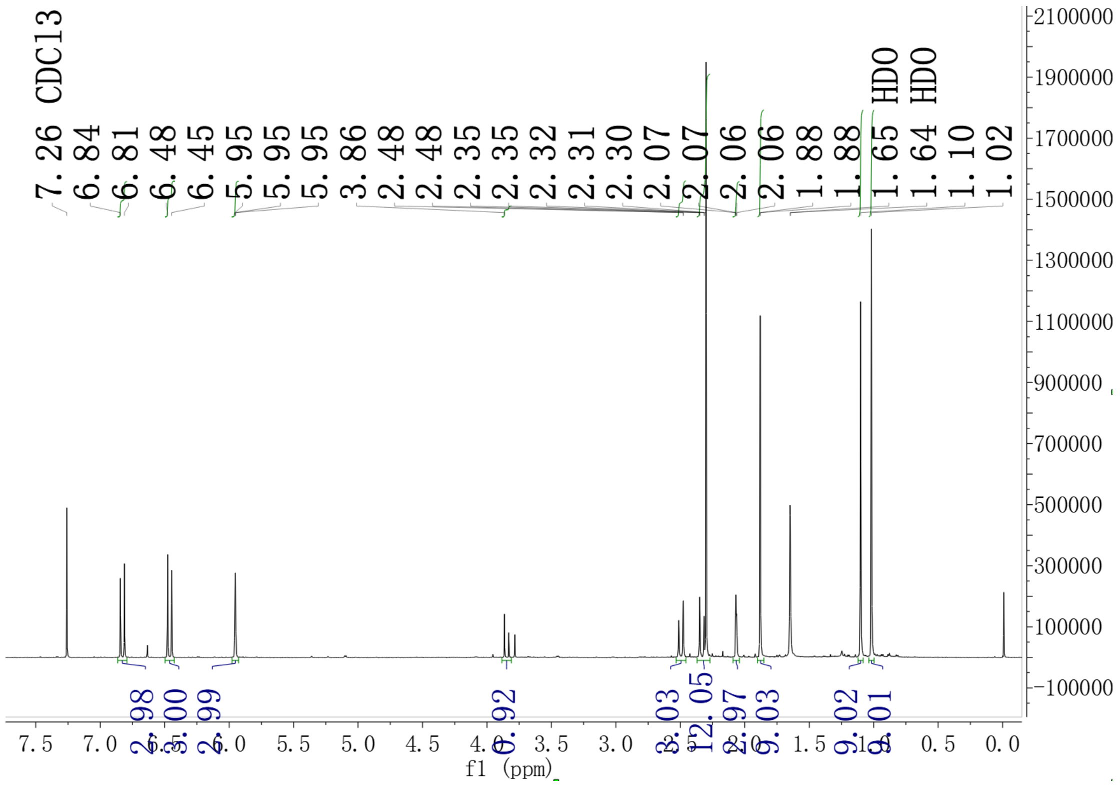

Figure S26. ^{1}H NMR ($CDCl_3$) spectrum of compound **9**

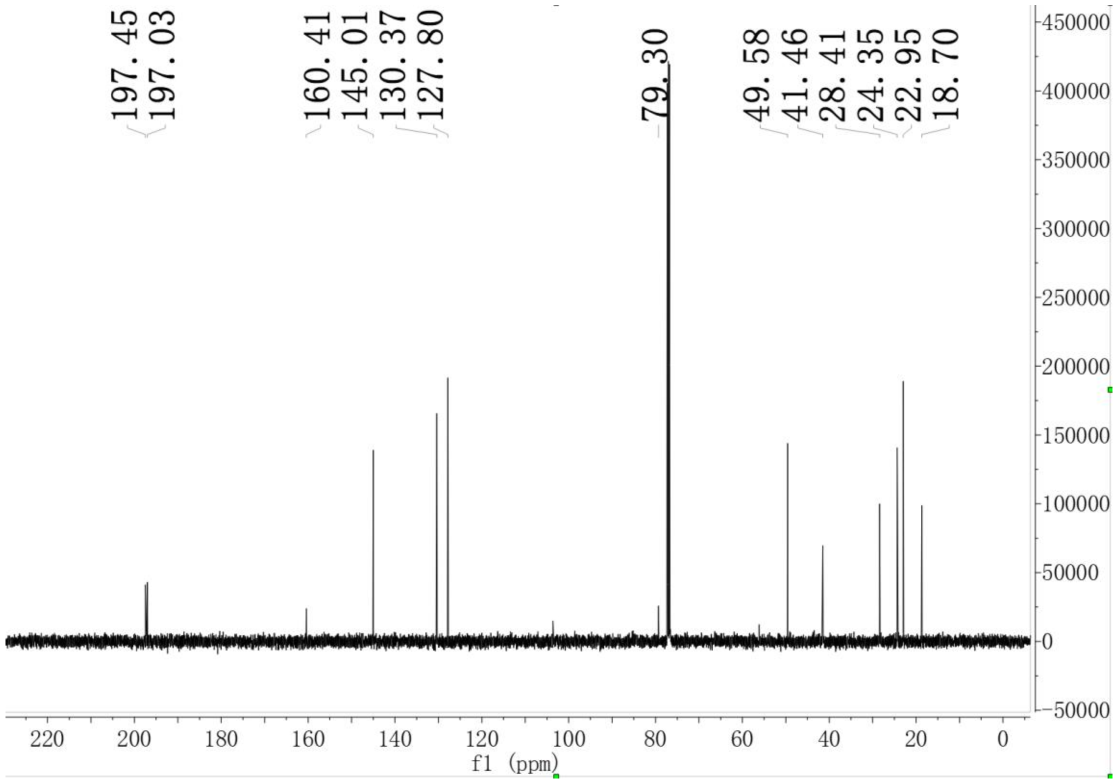

Figure S27.^{13}C NMR ($CDCl_3$) spectrum of compound **9**

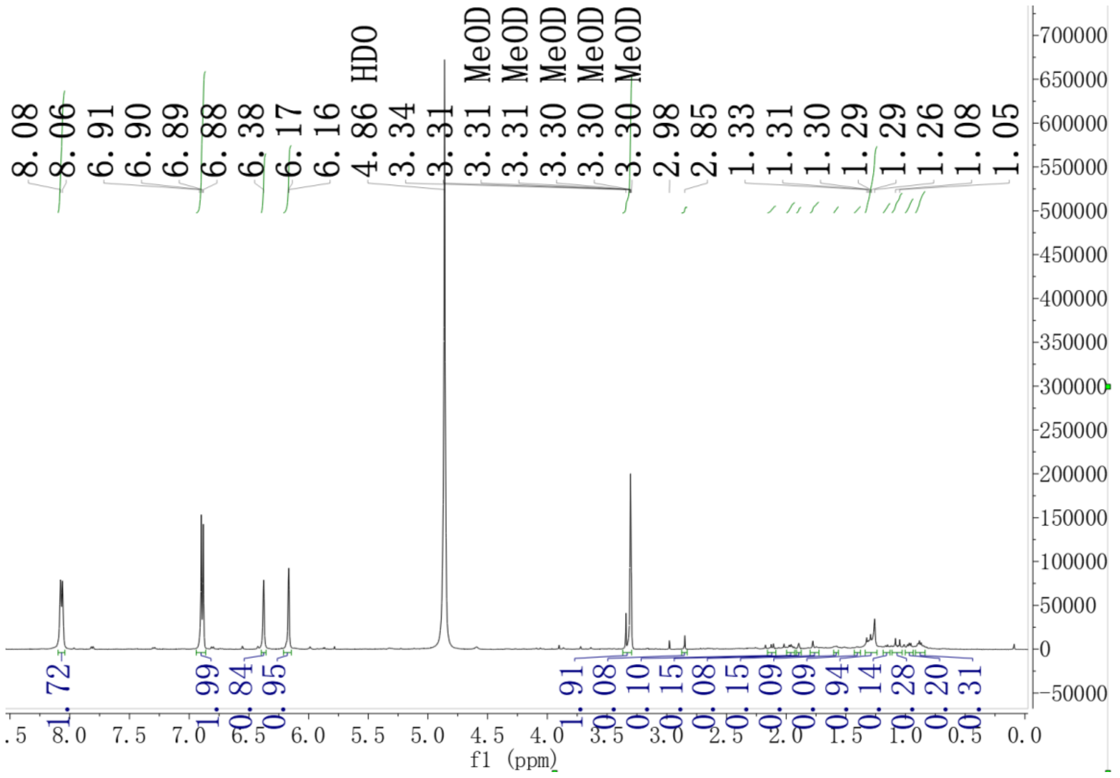

Figure S28. ^{1}H NMR (CD_3OD) spectrum of compound **10**

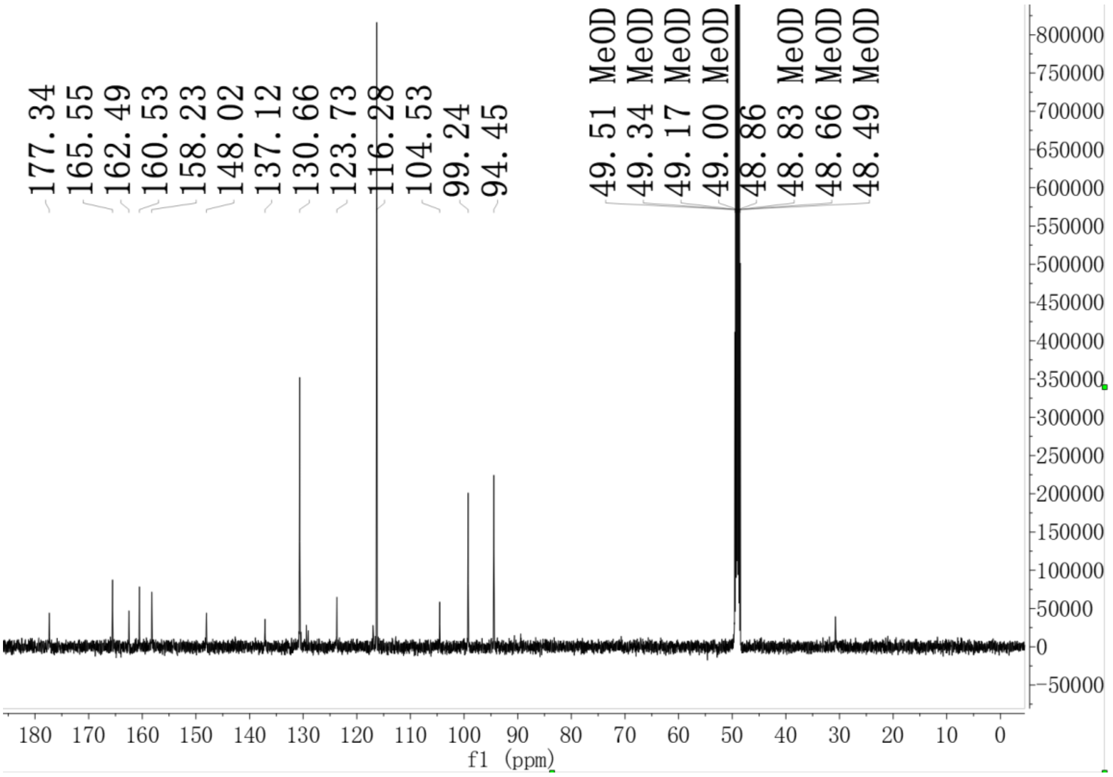

Figure S29. ^{13}C NMR (CD_3OD) spectrum of compound **10**

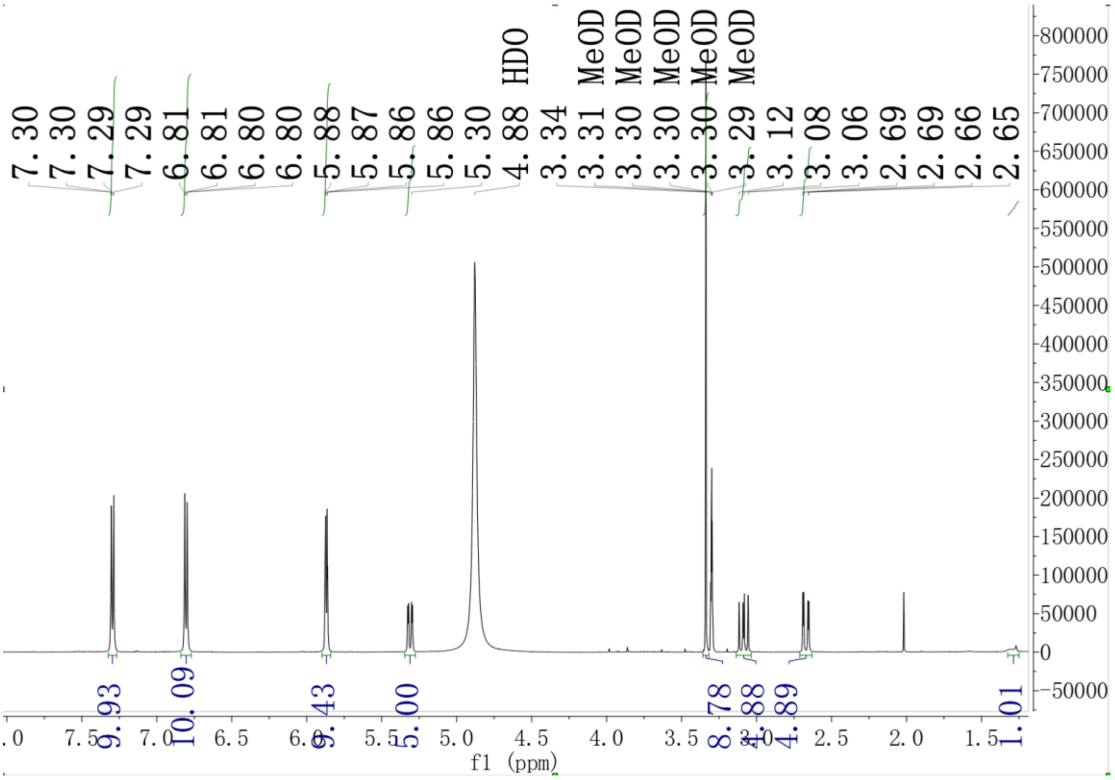

Figure S30. ^{1}H NMR (CD_3OD) spectrum of compound **11**

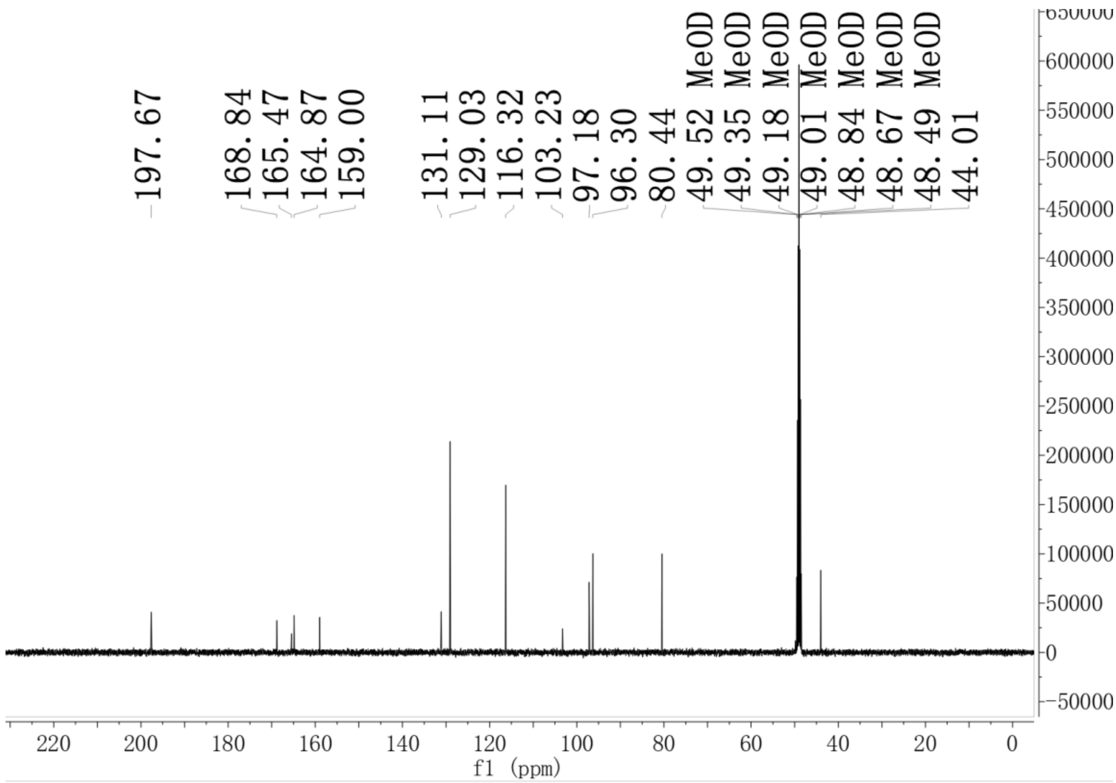

Figure S31. ^{13}C NMR (CD_3OD) spectrum of compound **11**

Table S1. Crystal data and structure refinement for compound **1**

Identification code	global	
Empirical formula	C20 H30 O3	
Formula weight	318.44	
Temperature	100(2) K	
Wavelength	1.54178 Å	
Crystal system	Monoclinic	
Space group	P 1 21 1	
Unit cell dimensions	a = 10.0464(12) Å	α= 90°.
	b = 9.5021(11) Å	β= 112.043(4)°.
	c = 10.0557(12) Å	γ = 90°.
Volume	889.77(18) $Å^3$	
Z	2	
Density (calculated)	1.189 Mg/m^3	
Absorption coefficient	0.614 mm^{-1}	
F(000)	348	
Crystal size	0.800 x 0.160 x 0.110 mm^3	
Theta range for data collection	4.74 to 72.24°.	
Index ranges	-12<=h<=12, -10<=k<=11, -12<=l<=12	
Reflections collected	17575	
Independent reflections	3416 [R(int) = 0.0553]	
Completeness to theta = 72.24°	99.7 %	
Absorption correction	Semi-empirical from equivalents	
Max. and min. transmission	0.94 and 0.71	
Refinement method	Full-matrix least-squares on F^2	
Data / restraints / parameters	3416 / 1 / 215	
Goodness-of-fit on F^2	1.048	
Final R indices [I>2sigma(I)]	R1 = 0.0341, wR2 = 0.0848	
R indices (all data)	R1 = 0.0359, wR2 = 0.0866	
Absolute structure parameter	0.03(9)	
Largest diff. peak and hole	0.155 and -0.167 e.$Å^{-3}$	

Table S2. Cytotoxicity data of compounds **1-11** at the concentration of 40 μM.

Name of compounds	HL-60		A549		SMMC-7721		MCF-7		SW480	
	Cell Inhibition(%)									
	Average	SD	Average	SD	Average	SD	Average	SD	Average	SD
**1**	34.32	2.94	12.18	0.36	28.32	1.89	37.31	1.98	32.05	3.50
**2**	32.71	0.90	9.03	2.86	22.76	1.96	11.86	0.72	15.19	0.62
**3**	22.73	2.70	27.71	1.27	19.41	2.60	22.27	0.84	14.30	2.39
**4**	79.64	1.84	88.08	0.44	79.42	2.27	80.52	0.69	93.76	2.85
**5**	0.00	2.46	30.24	0.93	20.66	1.47	27.13	1.80	19.78	2.09
**6**	15.82	2.57	6.89	0.83	1.60	2.00	0.56	2.14	8.70	1.08
**7**	17.73	2.08	27.41	0.52	21.26	1.55	10.72	1.32	31.98	0.86
**8**	33.57	0.90	9.98	0.59	11.38	1.66	5.32	3.11	5.78	1.78
**9**	12.96	2.07	-0.35	1.65	3.19	0.56	3.82	1.86	3.19	3.64
**10**	17.91	0.68	29.75	3.17	1.53	2.03	22.04	1.54	11.26	2.93
**11**	12.46	2.96	19.71	3.98	14.40	1.35	10.02	1.56	4.97	1.72
